# Synergistic T cell signaling by 41BB and CD28 is optimally achieved by membrane proximal positioning within parallel chimeric antigen receptors

**DOI:** 10.1016/j.xcrm.2021.100457

**Published:** 2021-12-21

**Authors:** Tamara Muliaditan, Leena Halim, Lynsey M. Whilding, Benjamin Draper, Daniela Y. Achkova, Fahima Kausar, Maya Glover, Natasha Bechman, Appitha Arulappu, Jenifer Sanchez, Katie R. Flaherty, Jana Obajdin, Kristiana Grigoriadis, Pierre Antoine, Daniel Larcombe-Young, Caroline M. Hull, Richard Buus, Peter Gordon, Anita Grigoriadis, David M. Davies, Anna Schurich, John Maher

**Affiliations:** 1 Leucid Bio Ltd., Guy’s Hospital, Great Maze Pond, London SE1 9RT, UK; 2 King’s College London, School of Cancer and Pharmaceutical Sciences, CAR Mechanics Lab, Guy’s Cancer Centre, Great Maze Pond, London SE1 9RT, UK; 3 King’s College London, Department of Infectious Diseases, School of Immunology and Microbial Sciences, Guy’s Hospital, Great Maze Pond, London SE1 9RT, UK; 4 King’s College London, School of Cancer and Pharmaceutical Sciences, Cancer Bioinformatics, Guy’s Cancer Centre, Great Maze Pond, London SE1 9RT, UK; 5 The Breast Cancer Now Toby Robins Research Centre at The Institute of Cancer Research, 237 Fulham Road, London SW3 6JB, UK; 6 Ralph Lauren Centre for Breast Cancer Research, Royal Marsden Hospital, Fulham Road, London SW3 6JJ, UK; 7 Department of Clinical Immunology and Allergy, King’s College Hospital NHS Foundation Trust, Denmark Hill, London SE5 9RS, UK; 8 Department of Immunology, Eastbourne Hospital, Kings Drive, Eastbourne, East Sussex BN21 2UD, UK

**Keywords:** chimeric antigen receptor, CAR T cells, co-stimulation, cancer, immunotherapy, parallel CAR, chimeric co-stimulatory receptor

## Abstract

Second generation (2G) chimeric antigen receptors (CARs) contain a CD28 or 41BB co-stimulatory endodomain and elicit remarkable efficacy in hematological malignancies. Third generation (3G) CARs extend this linear blueprint by fusing both co-stimulatory units in series. However, clinical impact has been muted despite compelling evidence that co-signaling by CD28 and 41BB can powerfully amplify natural immune responses. We postulate that effective dual co-stimulation requires juxta-membrane positioning of endodomain components within separate synthetic receptors. Consequently, we designed parallel (p)CARs in which a 2G (CD28+CD3ζ) CAR is co-expressed with a 41BB-containing chimeric co-stimulatory receptor. We demonstrate that the pCAR platform optimally harnesses synergistic and tumor-dependent co-stimulation to resist T cell exhaustion and senescence, sustaining proliferation, cytokine release, cytokine signaling, and metabolic fitness upon repeated stimulation. When engineered using targeting moieties of diverse composition, affinity, and specificity, pCAR T cells consistently elicit superior anti-tumor activity compared with T cells that express traditional linear CARs.

## Highlights

• Juxta-membrane localization is required for effective CAR-mediated co-stimulation
• To provide dual CD28 and 41BB co-stimulation, two fusion receptors are required
• The resulting parallel CAR configuration promotes functional persistence of T cells
• As a result, parallel CAR T cells mediate enhanced anti-tumor activity

## Graphical abstract

**Figure:**
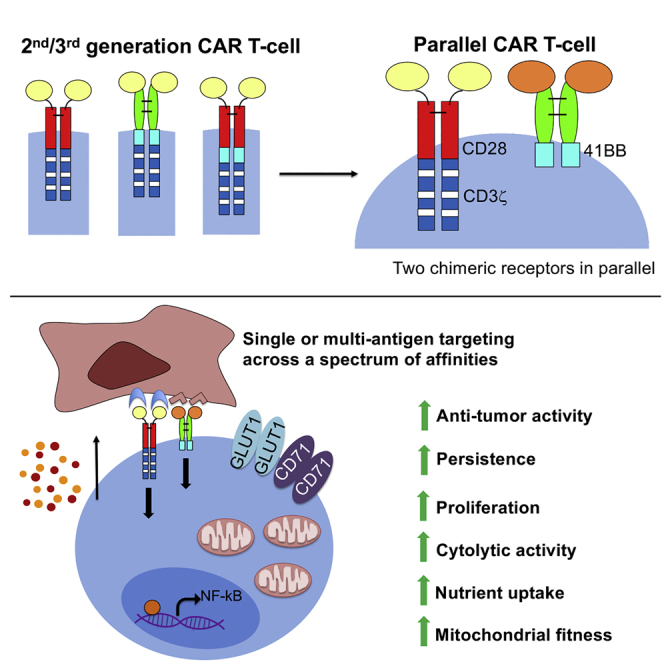

## Teaser

Dual co-stimulation by CD28 and 41BB contributes importantly to physiological immune responses, highlighting the desirability of harnessing these pathways to potentiate CAR T cell immunotherapy. Here, Muliaditan et al. show that this is achieved using a parallel CAR format, ensuring that both co-stimulatory domains sit in their natural membrane proximal location.

## Introduction

Chimeric antigen receptors (CARs) are modular synthetic units that re-direct lymphocyte specificity against cell surface targets. Conceived over 30 years ago,^1^ CAR technology was transformed from academic curiosity into groundbreaking cancer therapy with the demonstration that T cell receptor (TCR) and co-stimulatory signaling could be efficiently delivered via a single CD28+CD3ζ (28ζ) or 41BB+CD3ζ (BBζ) fusion.^2^^,^^3^ When these second generation (2G) CARs were evaluated in human T cells, anti-tumor activity proved markedly superior to first generation (1G) counterparts that provide TCR-like signaling alone.^4^^,^^5^ Infusion of 2G CAR T cells has achieved unprecedented efficacy against refractory B cell and plasma cell malignancy, emphasizing the crucial role of co-stimulation in therapeutic success. However, effectiveness against solid tumors remains inadequate, in large part due to tumor-induced T cell dysfunction.^6^

Given these considerations, it was logical to test whether potency could be augmented by insertion of an additional co-stimulatory element within a 2G CAR framework.^7^ Some studies reported increased efficacy of this third generation (3G) CAR approach in non-clinical testing.^7^, ^8^, ^9^, ^10^, ^11^, ^12^ However, this has not proven to be uniformly the case. When compared with 2G designs, some 3G CARs elicit borderline superiority,^13^, ^14^, ^15^ or even inferior anti-tumor activity.^16^^,^^17^ Ultimately, although CD28 and 41BB deliver amplifying co-stimulation,^18^, ^19^, ^20^ 3G CAR technology has not achieved meaningful clinical impact.

One potential reconciling factor relates to the location of signaling units in synthetic receptors. Juxta-membrane placement of CD28 is vital for its co-stimulatory function.^4^ However, the linear nature of the 3G CAR endodomain requires the positioning of one co-stimulatory module away from the membrane, meaning that access to downstream intermediates could be hindered by geographical or steric constraints. Signals that instruct T cell activation are naturally provided in *trans* by TCR/CD3 and one or more co-stimulatory receptors. Furthermore, co-expression of a CD28-containing 2G CAR with 41BB ligand (41BBL) achieves superior therapeutic function when compared with 3G fusion receptors.^21^^,^^22^ Accordingly, we hypothesized that a laterally configured CAR architecture would provide a more physiological platform to integrate such information.

Split chimeric receptors have been used to deliver “AND gated” activating and co-stimulatory signals,^23^^,^^24^ including an arrangement in which a 1G CAR was co-expressed with a CD28+41BB chimeric co-stimulatory receptor (CCR).^25^ However, the ability of such systems to provide synergistic dual co-stimulation remains unproven. Here, we demonstrate that membrane proximity is critical for effective co-stimulation by CD28 and 41BB and present a parallel (p)CAR arrangement that exploits this principle to consistently deliver superior anti-tumor activity.

## Results

### Design and expression of CARs and pCARs targeted against M-CSFR

**Figure 1:**
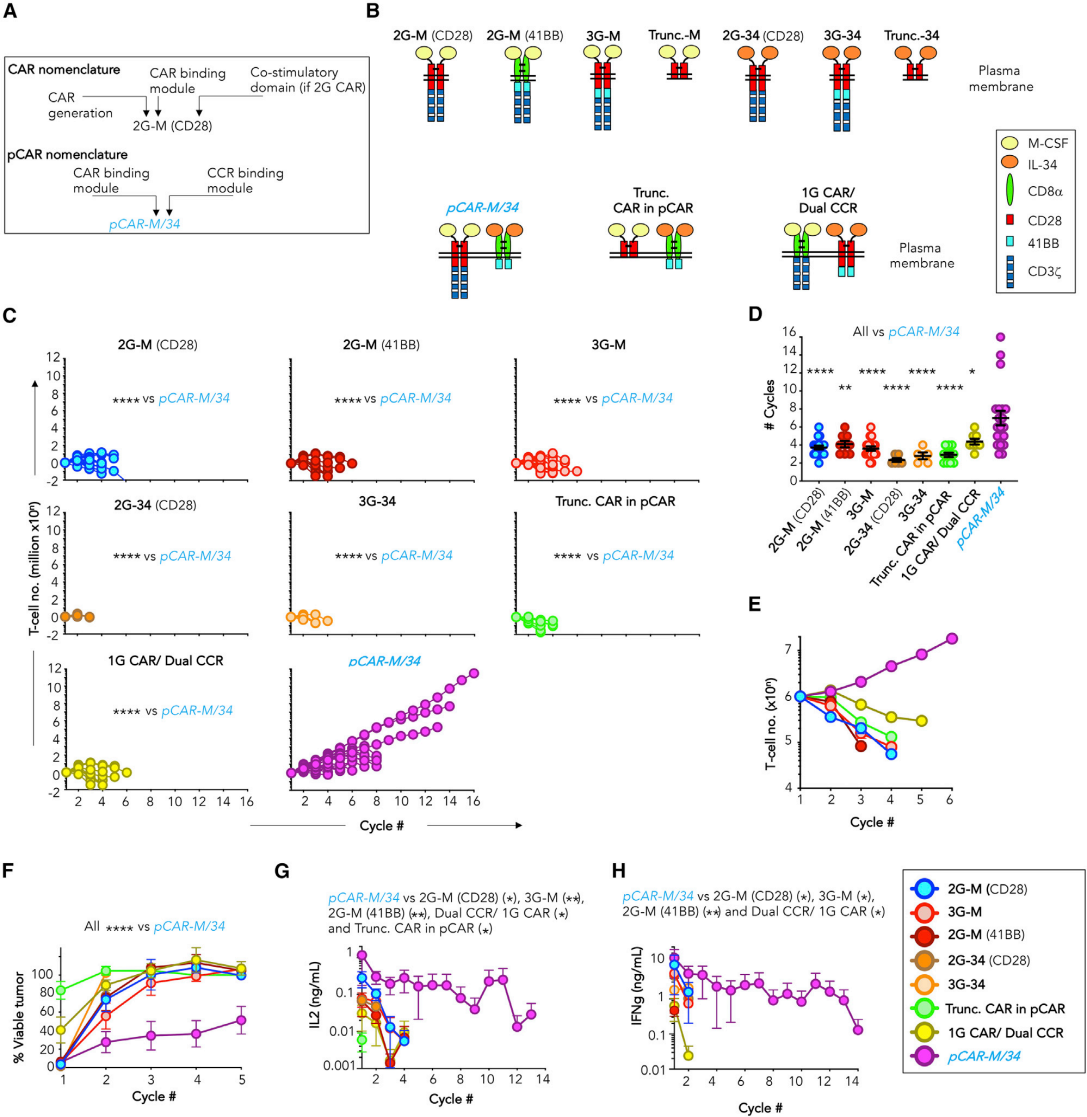
*In vitro* comparison of M-CSFR-targeted pCARs and linear CARs (A) Explanation of nomenclature. M, macrophage colony stimulating factor; 34, interleukin (IL)-34. Parallel CAR (pCAR) names are italicized throughout the text. (B) Cartoon structures illustrating M-CSFR-specific CARs, pCARs, and controls as follows: (1) an alternative binary configuration in which a 1G CAR (CD3ζ endodomain) was co-expressed with a CCR that contains fused CD28+41BB-signaling domains (1G CAR/dual CCR) and (2) an endodomain truncated pCAR that lacks 28ζ signaling sequences (trunc. CAR in pCAR). (C) The indicated engineered T cells (1 × 10^6^) were co-cultivated with T47D *FMS* cells without cytokine support. Each week, 1 × 10^6^ T cells were transferred to a new confluent monolayer. Spider plots enumerate T cell number over time (n = 5–20). Since data were not normally distributed, significance was determined using the Kruskal-Wallis test. Comparison with *pCAR-M/34* is shown. (D) Number of re-stimulation cycles in (C) from which T cells were retrieved. Statistical analysis was performed using one-way ANOVA, making comparison with *pCAR-M/34*. (E) “Mid-range” representative single-donor example in which 1 × 10^6^ M-CSFR re-targeted CAR or pCAR T cells were re-stimulated on T47D *FMS* tumor monolayers as described in (C) (mean ± SEM, n = 3 technical replicates). (F) Re-stimulation cultures in (C) were evaluated for tumor cell viability at 24 h (mean ± SEM), comparing *pCAR-M/34* (n = 10) with 2G-M (CD28) (n = 8), 2G-M (41BB) (n = 7), 3G-M (n = 8), 1G CAR/dual CCR (n = 5), 2G-34 (CD28) (n = 3), 3G-34 (n = 3), or Trunc. CAR in pCAR (n = 9). (G) IL-2 concentration in supernatants of re-stimulation cultures in (C) harvested 24 h after each stimulation (mean ± SEM, n = 5 [1G CAR/dual CCR], 6 [trunc. CAR in pCAR], 2 [2G-M (41BB)], 8 [2G-M (CD28), 3G-M], 10 [*pCAR-M/34*], and 3 [2G-34 (CD28), 3G-34]). Statistical analysis was performed using two-way ANOVA. (H) IFNγ concentration in supernatants of re-stimulation cultures in (C) harvested 24 h after each stimulation (mean ± SEM, n = 3 [1G CAR/dual CCR], 3 [2G-M (41BB)], 4 [2G-M (CD28), 3G-M], 4 [*pCAR-M/34*], and 3 [2G-34 (CD28), 3G-34]). Statistical analysis was performed using two-way ANOVA. ∗∗∗∗p < 0.0001; ∗∗∗p < 0.001; ∗∗p < 0.01; ∗p < 0.05. See Figures S1 and S2 and Table S1 for additional data.

Given the disappointing clinical impact of 3G CAR T cells, we hypothesized that a linear fusion of CD28 and 41BB fails to fully harness the potential for synergy between these distinct co-stimulatory receptors. To address this, we engineered a CAR construct in which CD28 and 41BB are expressed in *trans* within two separate receptors. By this means, each co-stimulatory unit is positioned adjacent to the plasma membrane, mimicking a more natural configuration. This approach was designated “pCAR” and consists of a 2G (CD28+CD3ζ) CAR co-expressed with a CCR. Nomenclature is explained in Figure 1A.

We selected the *FMS*-encoded macrophage colony stimulating factor receptor (M-CSFR) as a convenient model target to evaluate the pCAR system. M-CSFR is expressed in many cancers and binds two competing ligands with high (M-CSF; K_d_ of 34 pM) and very high (interleukin-34 [IL-34]; K_d_ of 1pM) affinity.^26^ A pCAR termed *pCAR-M/34* was engineered in which specificity of a 28ζ CAR was achieved using M-CSF, while 41BB CCR specificity was directed by IL-34 (Figure 1B). As controls, M-CSF and IL-34 were individually used to direct the specificity of 2G CARs (containing either CD28 or 41BB) and 3G CARs (CD28+41BB fusion) (Figure 1B). A control 1G CAR/dual CCR combination was also generated to further test the importance of membrane proximity in the delivery of co-stimulation. In this architecture,^25^ an M-CSF-directed 1G CAR was paired with a CD28+41BB-containing CCR targeted by IL-34 (Figure 1B). Finally, as a negative control, a signaling-deficient truncated pCAR was constructed (trunc. CAR in pCAR). This consisted of an M-CSF-targeted CAR in which the 28ζ endodomain had been removed and that was co-expressed with an IL-34-targeted 41BB CCR (Figure 1B). Consequently, T cell activation is not expected when this defective pCAR binds to M-CSFR. All of these M-CSFR-targeted fusion receptors were co-expressed with 4αβ, a chimeric cytokine receptor that couples the IL-4 receptor (R) α ectodomain to the transmembrane and endodomain of IL-2/15Rβ. 4αβ allows efficient IL-4-mediated enrichment of transduced cells, while fully preserving anti-tumor activity and type 1 polarity.^27^ Using this system, high-level cell surface expression of all fusion receptors was achieved (Figure S1).

### *pCAR-34/M* T cells outperform matched 2G and 3G counterparts *in vitro*

To test whether parallel placement of CD28 and 41BB co-stimulatory domains in membrane-proximal positions would yield synergistic signaling, we first evaluated the *in vitro* anti-tumor activity of *pCAR 34/M* T cells. In cancer, persistent antigen exposure leads to progressive T cell dysfunction,^28^ a process that can be modeled using tumor re-stimulation assays.^29^

To compare resistance to tumor-induced dysfunction, we iteratively restimulated CAR and pCAR T cells in the absence of cytokine by culture on T47D *FMS* or T47D monolayers, which respectively express or lack the M-CSFR target antigen. Each week, T cells were transferred to a new monolayer until they could no longer be retrieved. We made two striking observations in these experiments. First, when compared with a 2G (28ζ or BBζ) or 3G CAR, or the 1G CAR/dual CCR combination, *pCAR-M/34* T cells underwent significantly greater *FMS*-dependent expansion (Figure 1C) over more re-stimulation cycles (pooled data, Figure 1D; representative “mid-range” donor, Figure 1E). Target-dependent cytotoxicity (Figures 1F and S2) and release of both IL-2 (Figure 1G) and interferon γ (IFNγ) (Figure 1H) were also sustained for significantly longer in *pCAR-M/34* cultures. Findings were not due to differences in expression of CCR and/or CAR components that comprise these receptor systems (Figure S1). These data demonstrate that *pCAR-M/34* mediates increased resistance to T cell dysfunction, when compared with control CAR designs.

### pCAR-34/M provides effective dual co-stimulation through CD28 and 41BB

**Figure 2:**
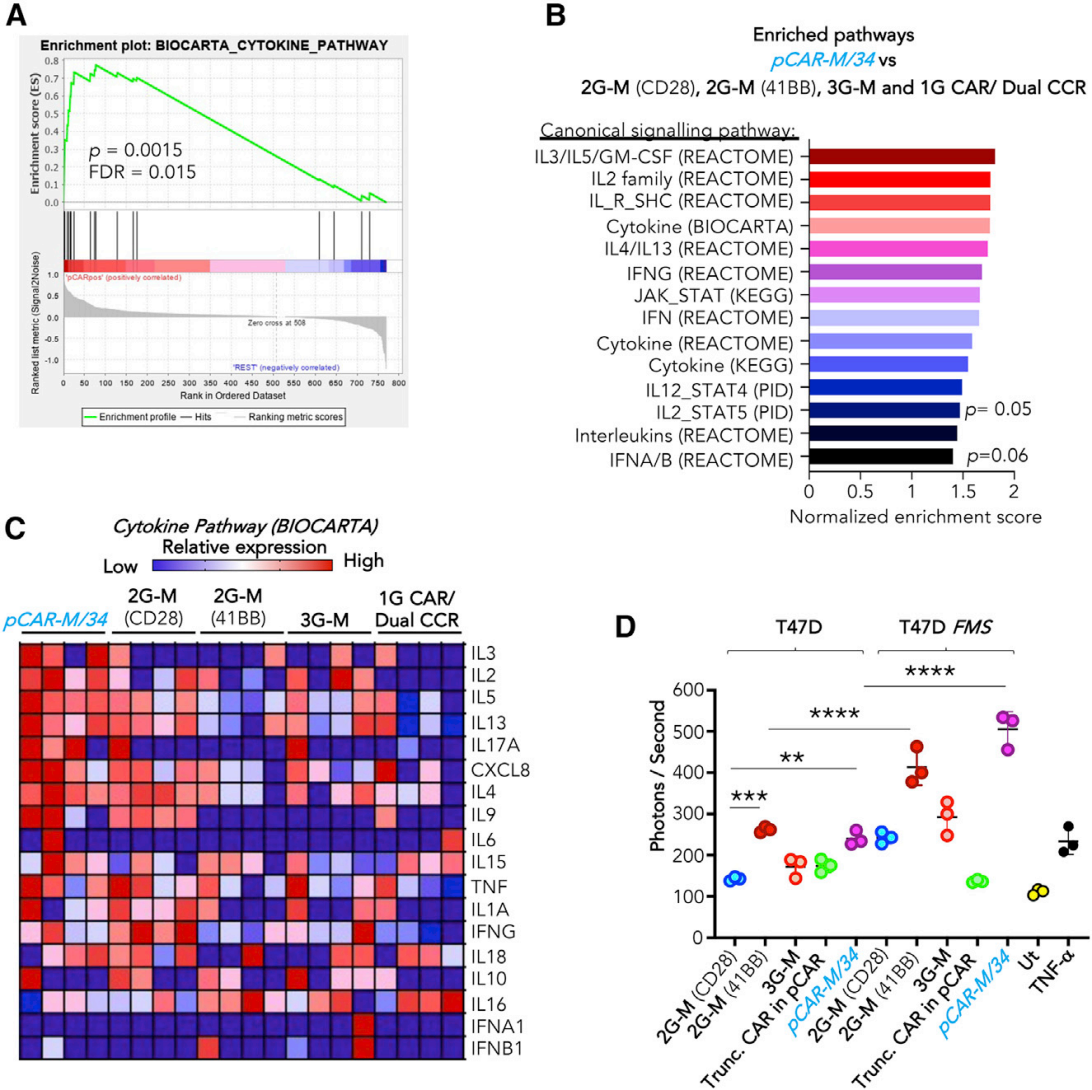
Investigation of co-stimulation delivered by *pCAR-M/34* (A) Carboxyfluorescein N-succinimidyl ester (CFSE)-labeled M-CSFR-specific CAR and pCAR T cells were stimulated for 24 h on T47D or T47D *FMS* tumor monolayers and then flow sorted prior to RNA extraction. Gene set enrichment analysis (GSEA) demonstrated significant cytokine pathway enrichment in *pCAR-M/34* T cells compared with all controls. (B) Enriched cytokine-signaling pathways (false discovery rate [FDR] < 0.25; p < 0.1) in *pCAR-M/34* pCAR T cells. p < 0.05 for all listed pathways, unless indicated otherwise. (C) “Blue pink o’gram” heatmap of cytokine gene expression in *pCAR-M/34* and control T cell populations following stimulation on T47D *FMS* tumor monolayers. (D) Engineered Jurkat NF-κB reporter cells were co-cultured with T47D or T47D *FMS* cells for 5 h. Cell lysates were then analyzed for luciferase activity (mean ± SD, n = 3). Effect of tumor necrosis factor alpha (TNF-α) is shown as positive control. M-CSFR-specific CAR and pCAR T cells were re-stimulated each week as described in Figure 2C. See Figure S3 for additional data.

Next, we characterized the ability of *pCAR-M/34* to deliver dual co-stimulation. CD28 co-stimulation manifests with increased cytokine release and proliferation.^2^^,^^4^ Delivery of these attributes by *pCAR-M/34* is indicated in the functional experiments shown in Figures 1C–1E, 1G, and 1H. To provide further confirmation, NanoString analysis was performed after overnight co-culture of pCAR and CAR T cells (Figure S3A) with T47D *FMS* or T47D monolayers (data deposited at Gene Expression Omnibus [GEO]: GSE186557). Although we noted donor-to-donor variability, activated *pCAR-M/34* T cells displayed enriched gene expression linked to multiple cytokine-signaling pathways (Figures 2A–2C). These findings emphasize that strong CD28-mediated signaling is evident in these pCAR T cells.

41BB co-stimulation promotes nuclear factor κB (NF-κB) activation,^30^ which can be conveniently quantified using Jurkat cells in which firefly luciferase (ffLuc) expression is placed under the transcriptional control of the NF-κB promoter. Jurkat reporter cells were engineered to express *pCAR-M/34* and 2G or 3G control CARs and then added to T47D or T47D *FMS* monolayers for 5 h (Figure 2D). 2G-M (41BB) and *pCAR-M/34* Jurkat T cells both exhibited significantly heightened basal NF-κB activity, consistent with ligand-independent tonic signaling by the 41BB-containing receptor. When stimulated on T47D *FMS* monolayers, this signal increased greatly in both cases, demonstrating that strong ligand-dependent 41BB co-stimulation had occurred. By contrast, activation of the 2G-M (CD28) CAR triggered a marginal and non-significant increment in NF-κB activity, consistent with a lack of 41BB signaling. Importantly, a similar profile was seen with the 3G-M CAR, indicative of deficient 41BB co-stimulation. These data show that the *pCAR-M/34*, unlike the 3G-M CAR, effectively harnesses both CD28 and 41BB co-stimulatory pathways.

### Dual co-stimulation by *pCAR-M/34* counteracts tumor-induced T cell dysfunction and induces metabolic remodeling

**Figure 3:**
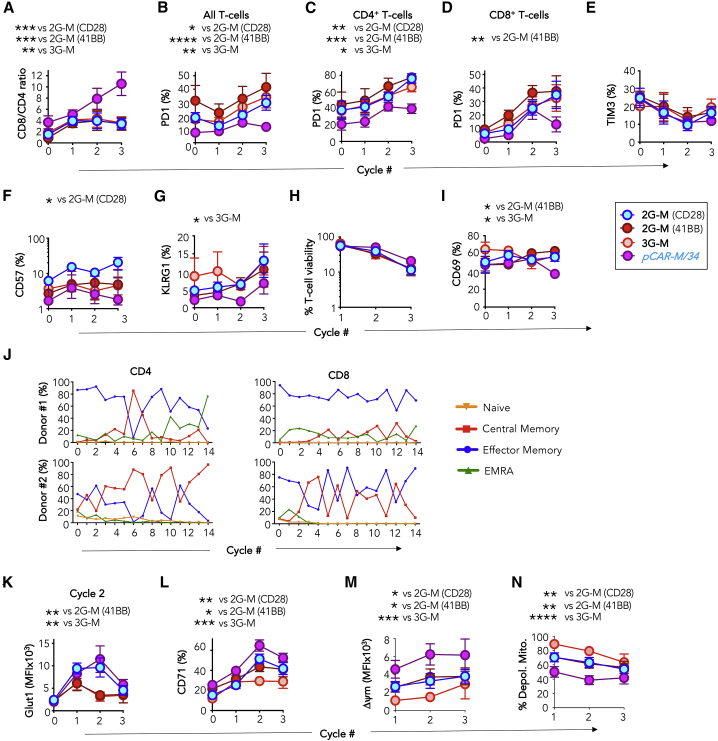
Re-stimulated *pCAR-M/34* T cells downregulate pathways that favor tumor-induced dysfunction and undergo metabolic remodeling (A–I) The indicated engineered T cells (1 × 10^6^) were iteratively re-stimulated on T47D *FMS* monolayers without cytokine support. Each week, 1 × 10^6^ T cells were transferred to a new confluent monolayer. Twenty-four hours after the initiation of each stimulation cycle, T cells were analyzed for the following attributes. All statistical analysis was performed by two-way ANOVA with multiple comparisons. ∗p < 0.05; ∗∗p < 0.01; ∗∗∗p < 0.001; ∗∗∗p < 0.0001, making comparison in each case with *pCAR-M/34* T cells. (A) CD8/CD4 ratio (mean ± SEM, n = 4–12). (B) %PD1 (all T cells; mean ± SEM, n = 4–22). (C) %PD1 on CD4^+^ T cell subset (mean ± SEM, n = 4) (D) %PD1 on CD8^+^ T cell subset (mean ± SEM, n = 4). (E) %TIM-3 (mean ± SEM, n = 2–7). (F) %CD57 (median ± interquartile range; n = 4–16). (G) %KLRG1 (mean ± SEM, n = 4). (H) % viability (mean ± SEM, n = 4–10). (I) %CD69 (mean ± SEM, n = 4). (J) *pCAR-M/34* T cells from two independent donors were iteratively stimulated once weekly on T47D *FMS* monolayers. Prior to each stimulation cycle, cells were stained with CD45RO, CCR7, CD4, and CD8 and assigned as naive (CD45RO^−^ CCR7^+^), central memory (CD45RO^+^ CCR7^+^), effector memory (CD45RO^+^ CCR7^−^), or EMRA (CCR7^−^, CD45RO^−^) within the CD4^+^ or CD8^+^ subset. (K–N) The indicated engineered T cells (1 × 10^6^) were iteratively re-stimulated on T47D *FMS* monolayers without cytokine support. Each week, 1 × 10^6^ T cells were transferred to a new confluent monolayer. Twenty-four hours after the initiation of each stimulation cycle, T cells were analyzed for the following attributes. All statistical analysis was performed by two-way ANOVA with multiple comparisons. ∗p < 0.05; ∗∗p < 0.01; ∗∗∗p < 0.001; ∗∗∗p < 0.0001, making comparison in each case with *pCAR-M/34* T cells. (K) Glut1 mean fluorescence intensity (MFI) (mean ± SEM, n = 3–16). Please note that 2G-M (41BB) and 3G-M plots are closely aligned. (L) %CD71 (mean ± SEM, n = 4–14). (M) Mitochondrial membrane potential (Δψm; MFI; mean ± SEM, n = 4-7). (N) %Depol(arized) mito(chondria) (Q; mean ± SEM, n = 4–7).

Next, we evaluated the impact of *pCAR-M/34* signaling on tumor-induced T cell dysfunction and metabolic fitness. Key underlying mechanisms of T cell dysfunction are exhaustion, senescence, activation-induced cell death (AICD), and accelerated differentiation. Re-stimulated *pCAR-M/34* T cells underwent progressive and significantly greater CD8^+^ T cell expansion (Figure 3A) while maintaining significantly lower anti-human CD279 (PD1) expression on both CD4^+^ and CD8^+^ T cell subsets (Figures 3B–3D). Tim3 levels matched those of controls (Figure 3E). These data are consistent with reduced exhaustion of *pCAR-M/34* T cells due to improved co-stimulation.

Senescence is also a common feature of intra-tumoral T cells.^31^ Expression of the senescence markers CD57 (Figure 3F) and KLRG1 (Figure 3G) were both reduced in re-stimulated *pCAR-M/34* T cell cultures. Although CD28 and 41BB co-stimulation can reduce AICD,^32^ CAR T cells are particularly susceptible to this process.^33^ In keeping with this, we observed a progressive increase in AICD of re-stimulated CAR T cells. However, this was slightly attenuated in *pCAR-M/34* T cells (Figure 3H), accompanied by reduced expression of the CD69 activation marker (Figure 3I).

Co-stimulation also plays a key role in the regulation of T cell differentiation and memory formation.^34^ Notably, periodic enrichment of central memory T cells was observed in re-stimulated *pCAR-M/34* cultures over prolonged tumor re-stimulation (Figure 3J). This may simulate the three phases seen during T cell immune responses in which antigen-specific cells undergo expansion followed by contraction and memory formation.

Finally, signaling by both CD28^35^ and 41BB^36^ facilitates metabolic reprogramming that could promote greater resistance of *pCAR-M/34* T cells to tumor-induced dysfunction. In support of this, re-stimulated *pCAR-M/34* T cells maintained high levels of the key nutrient transporters, glucose transporter 1 (Glut1) (Figure 3K) and transferrin receptor CD71 (Figure 3L). These data accord with the importance of activation-induced glucose and iron uptake in T cell proliferation and mitochondrial function, respectively.^37^^,^^38^ We and others have shown that T cell exhaustion is accompanied by loss of mitochondrial function.^29^^,^^39^^,^^40^ In line with their increased functionality, re-stimulated *pCAR-M/34* T cells maintained higher mitochondrial membrane potential (Δψm; Figure 3M) and harbored less depolarized mitochondria (Figure 3N). This phenotype has also been associated with superior T cell survival and function in the tumor microenvironment (TME).^40^ Together, these data indicate that dual CD28 and 41BB co-stimulation provided by *pCAR-M/34* results in metabolic remodeling and enhanced resistance to tumor-induced T cell dysfunction.

### *pCAR-M/34* T cells show superior therapeutic activity against an M-CSFR^+^ lymphoma xenograft

**Figure 4:**
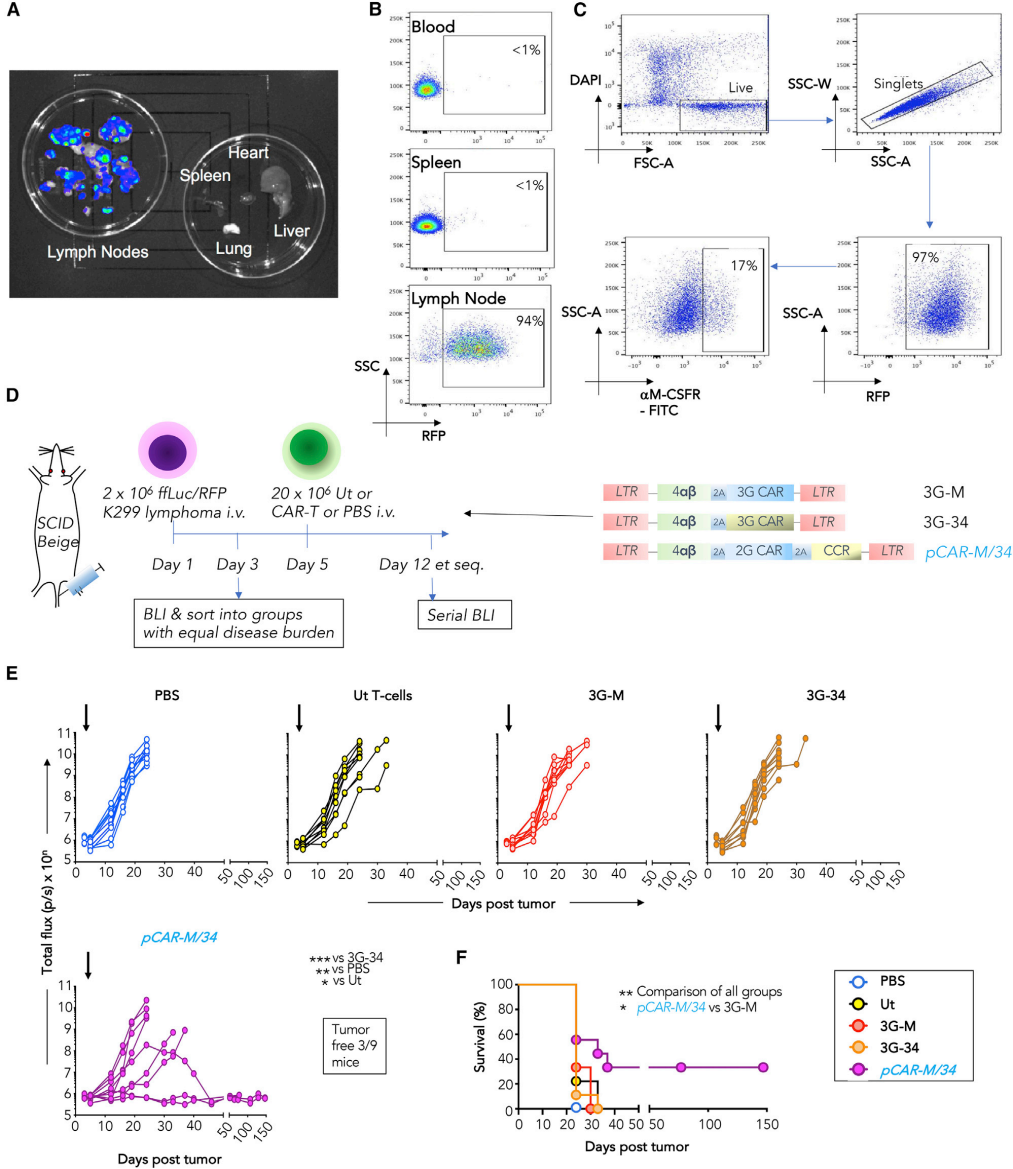
M-CSFR re-targeted pCAR T cells elicit superior anti-lymphoma activity *in vivo* (A) To establish a xenograft model of M-CSFR^+^ anaplastic cell lymphoma, severe combined immunodeficiency (SCID) Beige mice were inoculated i.v. with 2 × 10^6^ red fluorescent protein (RFP)/ffLuc^+^ K299 cells. After 24 days, luciferin was administered prior to culling of mice and major organs analyzed using BLI. (B) Blood, spleen, and lymph nodes were analyzed for RFP^+^ tumor cells by flow cytometry. (C) Lymph node tumors were mechanically disaggregated to yield a single-cell suspension, which was stained with αM-CSFR antibody. (D) To test *in vivo* anti-tumor activity of M-CSFR re-targeted T cells, SCID Beige mice (n = 9 per group) were inoculated i.v. with 2 × 10^6^ ffLuc/RFP^+^ K299 cells. On day 5, mice were treated i.v. with 20 × 10^6^ of the indicated CAR, pCAR, or untransduced (UT) T cells or PBS. (E) Tumor burden in individual mice following treatment as described in (D) was monitored by BLI. Day of T cell injection is indicated by the arrow. Data are pooled from two experiments in which CAR T cells were prepared from different donors (n = 5 per group experiment 1; n = 4 per group experiment 2). Statistical analysis was performed using two-way ANOVA, comparing the *pCAR-M/34* group with the indicated CARs on day 24 post tumor inoculation. (F) Survival curve of mice. Statistical analysis was performed using a log rank (Mantel-Cox) test, comparing the indicated groups. ∗p < 0.05; ∗∗p < 0.01; ∗∗∗p < 0.001.

We next evaluated *in vivo* anti-tumor activity of *pCAR-M/34* T cells against a challenging Karpas (K)299 anaplastic lymphoma xenograft that undergoes rapid lymphatic dissemination (Figures 4A and 4B) and expresses low levels of the M-CSFR (Figure 4C). Mice with established disease were treated intravenously (i.v.) with *pCAR-M/34* T cells (experimental design, Figure 4D), making comparison with 3G CAR T cells in which specificity was conferred by either M-CSF or IL-34. Untransduced (UT) T cells and PBS served as additional controls. While 3G CAR T cells demonstrated no efficacy, 3 of 9 mice that received *pCAR-M/34* T cells achieved sustained tumor control, while disease progression was delayed in 2 further mice (Figure 4E), leading to significant prolongation of survival (Figure 4F). These data reinforce the superior *in vivo* anti-tumor activity of the *pCAR-M/34* format compared with linear CAR counterparts that contain identical co-stimulatory units.

### pCAR T cells with dual target specificity achieve superior potency while maintaining targeting precision

In designing binary systems such as pCAR, it may be desirable to enhance potency while maintaining tumor specificity by directing each receptor component against two non-competing antigens.^24^ To ensure safety, effector function should be absolutely dependent on engagement of a tumor-selective CAR target, permitting the inclusion of a less stringently tumor-specific CCR. We tested this by engineering T cells to express a pCAR in which a mucin 1 (MUC1)-specific 2G (28ζ) CAR was paired with a 41BB CCR that binds multiple ErbB dimers. While MUC1 has been ranked as the highest priority cell-surface cancer antigen,^41^ CAR T cell targeting of ErbB dimers has demonstrated great potency, but limited tumor specificity.^42^^,^^43^

**Figure 5:**
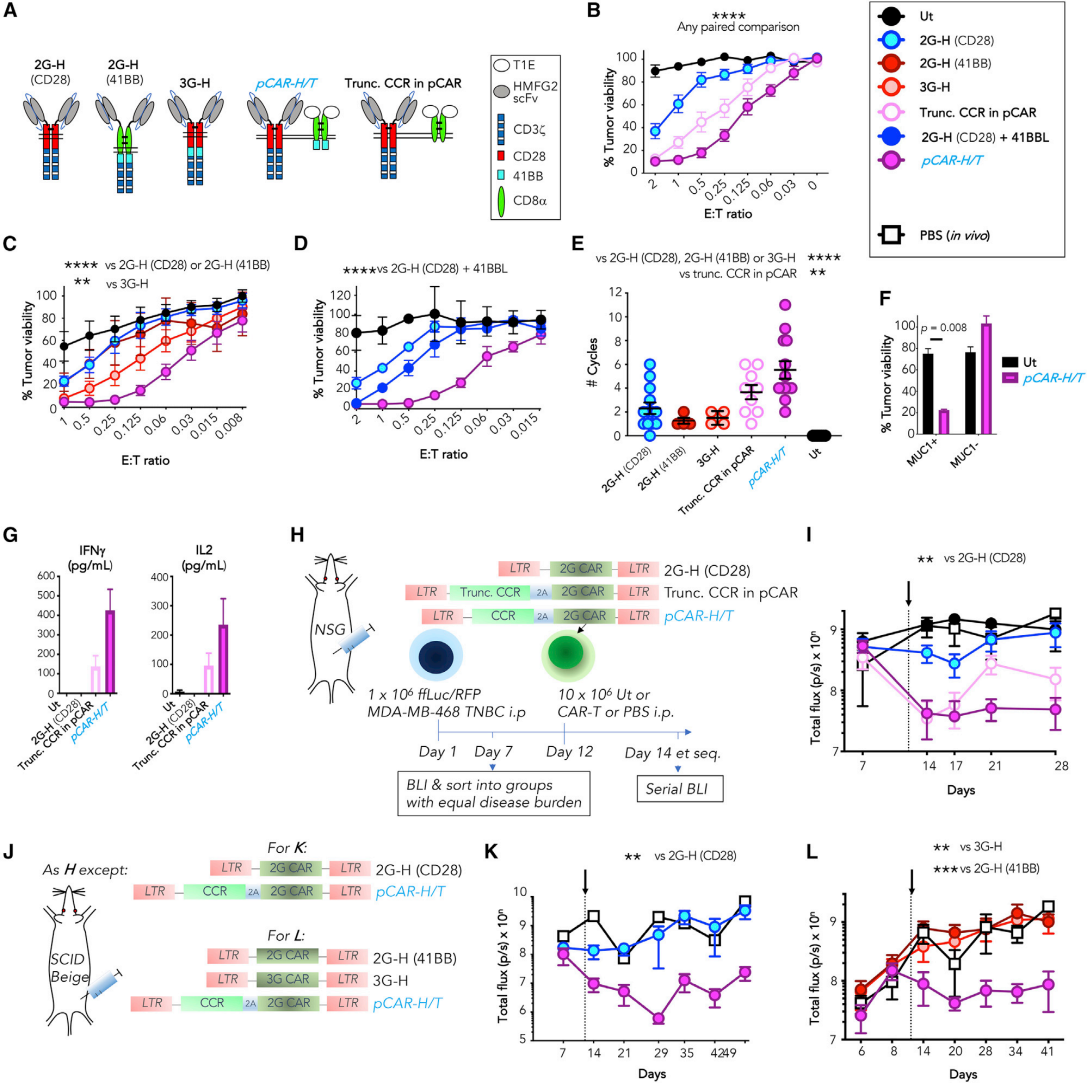
Co-targeting of MUC1 and ErbB dimers using pCAR T cells (A) Cartoon structure of MUC1-specific CARs in addition to a pCAR and truncated pCAR control that co-target MUC1 and ErbB dimers. (B) T cells were engineered to express *pCAR-T/H* or controls and co-cultured at the indicated E:T ratio with MDA-MB-468 tumor cells, without exogenous cytokine. Target viability was assessed after 72 h (mean ± SEM, n = 6). Statistical analysis shown in this figure was performed by two-way ANOVA with multiple comparisons and compares individual CARs across all E:T ratios. ∗p < 0.05; ∗∗p < 0.01; ∗∗∗p < 0.001; ∗∗∗p < 0.0001. (C) Cytotoxicity assays making comparison with the indicated controls, performed as described in (B) (mean ± SEM, n = 10). Significance compares the indicated CAR to *pCAR-H/T*. (D) Cytotoxicity assays making comparison with the indicated controls, performed as described in (B). Data shown are mean ± SEM, of n = 5 (2G-H (CD28)), 6 (*pCAR-H/T* and 2G-H (CD28) + 41BBL), or 2 (UT) donors. Significance compares the indicated CAR to *pCAR-H/T*. (E) T cells were re-stimulated twice per week by co-culture with MDA-MB-468 tumor cells (E:T ratio 1). Data show the mean ± SEM of the number of re-stimulation cycles in which T cells could be retrieved. Significance compares the indicated CAR to *pCAR-H/T*. (F) pCAR or UT T cells were co-cultivated for 48 h with ffLuc/RFP^+^ MDA-MB-435 HER2 tumor cells (MUC1−) or a derivative that overexpresses MUC1 (MUC1+). Residual tumor viability was determined by luciferase assay (E:T ratio 1; mean ± SEM, n = 2). (G) Supernatant was collected from cultures shown in (B) (E:T ratio 0.5) after 24 h and analyzed for the indicated cytokines (mean ± SEM, n = 6). Differences are not statistically significant. (H) NOD SCID common gamma chain null (NSG) mice were inoculated intraperitoneally (i.p.) with 1 × 10^6^ RFP/ffLuc^+^ MDA-MB-468 tumor cells. After 12 days, 10 × 10^6^ T cells that express the indicated CAR or pCAR were injected i.p., making comparison with PBS. (I) Tumor burden following treatment as described in (H) was monitored by serial BLI (mean ± SEM, n = 5 mice). Day of T cell injection is indicated by the arrow. Significance compares the indicated CAR to *pCAR-H/T*. (J) SCID Beige mice were inoculated with tumor as described in (H). After 12 days, mice were treated i.p. with T cells engineered to express the indicated CARs/pCARs. (K) Tumor burden was monitored following treatment as described in (J) by serial BLI (n = 3 [*pT/H* and H-2] or 1 [PBS]). Day of T cell injection is indicated by the arrow. Significance compares the indicated CAR to *pCAR-H/T*. (L) As in (K) (mean ± SEM, n = 3 per group). Significance compares the indicated CAR to *pCAR-H/T*. See Figure S4 for additional data.

Previously, we used an human milk fat globulin (HMFG)2 single chain antibody fragment (scFv) to engineer a MUC1-specific 2G CAR,^13^ here called 2G-H (CD28) (Figure 5A). To engineer the pCAR, the 2G-H (CD28) CAR was co-expressed with a 41BB-containing CCR that binds eight distinct ErbB homo- and heterodimers (Figure 5A). Target specificity of the CCR was achieved using a promiscuous ErbB ligand termed T1E, which is a chimera derived from transforming growth factor α and epidermal growth factor.^42^^,^^43^ The resulting construct was referred to as *pCAR-H/T* (i.e., HMFG2-targeted CAR and T1E-targeted CCR). To serve as additional controls, the 2G-H (41BB) and 3G-H CARs were designed. We also constructed a truncated pCAR control (trunc. CCR in pCAR) in which the 2G-H (CD28) CAR was co-expressed with a T1E-targeted CCR that lacked the 41BB co-stimulatory endodomain and thus was signaling defective (Figure 5A). Expression of these CARs and pCARs in human T cells is shown in Figure S4A.

To compare function of these constructs, cytotoxicity assays were performed using MDA-MB-468 triple negative breast cancer (TNBC) cells, which naturally express both MUC1 and ErbB dimers. While 2G CAR T cells only exerted strong cytolytic activity at a high effector to target (E:T) ratio, *pCAR-H/T* T cells achieved significantly enhanced tumor cell killing at low E:T ratios (Figure 5B). Cytotoxic activity of *pCAR-H/T* T cells also significantly exceeded that of both 2G-H (41BB) and 3G-H T cells (Figure 5C) or the combination of 2G-H (CD28) and 41BBL (Figure 5D).^21^ When tested in tumor re-stimulation assays, *pCAR-H/T* T cells maintained cytolytic activity over significantly more cycles than all controls (Figure 5E). Of particular importance, cytotoxic activity of *pCAR-H/T* T cells remained strictly MUC1 dependent. This is indicated by lack of cytotoxicity against MUC1-negative MDA-MB-435 human epidermal growth factor receptor 2 (HER2) cells that express HER2-containing ErbB dimers that are bound by the T1E peptide^42^ (Figure 5F).

Cytokine production by 2G 2G-H (CD28) CAR T cells was poor. By contrast, activated *pCAR-H/T* T cells produced higher levels of IL-2 and IFNγ (Figure 5G). Notably, the trunc. CCR in pCAR control that could also bind MUC1 and ErbB dimers elicited intermediate cytotoxicity (Figure 5B), tumor re-stimulation potential (Figure 5E), and cytokine release (Figure 5F). Since this control lacks a 41BB endodomain, this indicates that the CCR component of *pCAR H/T* enhances function both through signaling-dependent and -independent mechanisms.

Next, *in vivo* anti-tumor activity was compared. Superior anti-tumor activity of *pCAR H/T* cells was observed in mice with an established MDA-MB-468 xenograft (Figures 5H–5L) or a HER2-overexpressing derivative (Figures S4B–S4D), when compared with 2G (CD28 [Figures 5I, 5K, and S4C]; 41BB [Figure 5L]) or 3G controls (Figure 5L). Once again, T cells that expressed the trunc. CCR in pCAR control achieved intermediate anti-tumor activity, in keeping with signaling-independent activity of the CCR (Figure 5I).

The T1E peptide used to target CCR specificity in *pCAR-H/T* binds to murine ErbB dimers efficiently, meaning that CAR T cells containing T1E can mediate significant and sometimes lethal toxicity in mice.^43^^,^^44^ Nonetheless, no clinical evidence of *in vivo* toxicity was observed in any of these experiments. Together with the stringent dependence of the *pH/T* pCAR cytolytic activity on MUC1 engagement (Figure 5F), this demonstrates that absolute dependence on the CAR target is indeed maintained in the pCAR arrangement. Importantly, anti-tumor activity is boosted when both CAR and CCR targets are co-engaged.^24^^,^^25^ Together, these attributes render pCAR T cells better suited to function in the challenging microenvironment of a solid tumor, given the paucity of truly disease-specific targets that are available.

### pCAR T cells mediate enhanced recall responses and durability of *in vivo* anti-tumor activity

**Figure 6:**
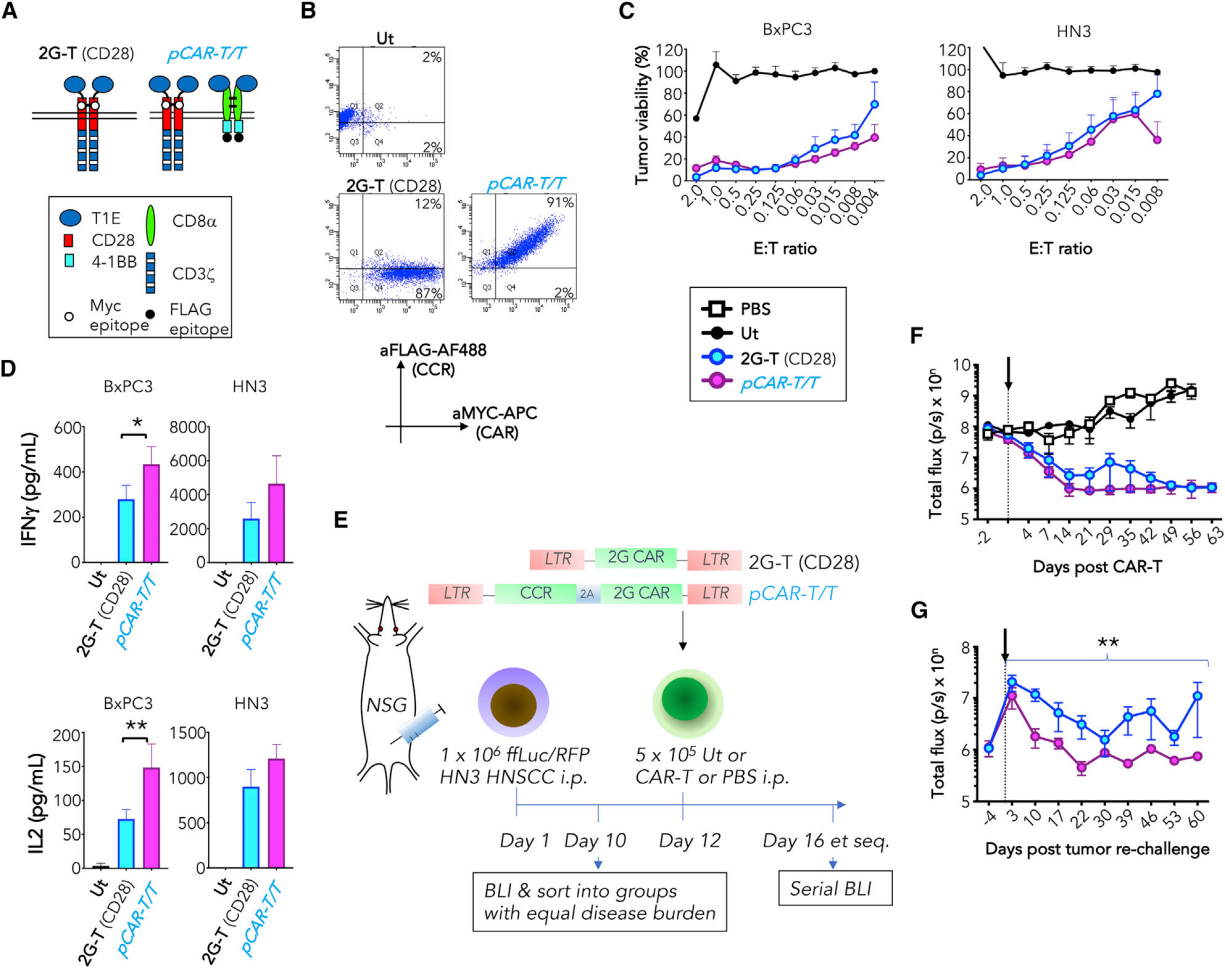
pCAR T cells demonstrate sustained functional persistence *in vivo* (A) Cartoon structure of panErbB-specific 2G CAR (2G-T (CD28)) and pCAR (*pCAR-T/T*). (B) Expression of panErbB-specific pCARs in human T cells was determined by flow cytometry using antibodies directed against embedded epitope tags within the CAR and CCR component (representative of n = 3 replicates). (C) T cells were engineered to express *pCAR-T/T* or 2G-T (CD28) control and were cultured at the indicated E:T ratio with BxPC3 or HN3 tumor cells, without cytokine support. Tumor cell viability was assessed after 72 h (mean ± SEM, n = 7), making comparison with UT T cells. (D) Supernatant was collected after 24 h (1:1 E:T ratio) and analyzed for the indicated cytokines (mean ± SEM, n = 7 [BxPC3 IFNγ], 11 [BxPC3 IL2], 9 [HN3 IFNγ], or 10 [HN3 IL2]). Statistical analysis was performed using a paired t test. (E) NSG mice were inoculated i.p. with 1 × 10^6^ RFP/ffLuc^+^ HN3 tumor cells. After 12 days, 5 × 10^5^ T cells that express the indicated CAR or pCAR were injected i.p., making comparison with UT T cells and PBS. (F) Tumor burden was monitored following treatment as described in (E) using BLI (mean ± SEM, n = 5 mice per group). Day of T cell injection is indicated by the arrow. (G) Tumor-free mice were re-challenged by i.p. injection of 1 × 10^6^ RFP/ffLuc^+^ HN3 tumor cells on day 63 post initial tumor inoculation (arrowed). Tumor burden was monitored thereafter using BLI (mean ± SEM, n = 5 mice per group). Statistical analysis was performed using a Mann-Whitney test. ∗p < 0.05; ∗∗p < 0.01; ∗∗∗p < 0.001; ∗∗∗p < 0.0001.

We hypothesized that effective dual CD28 and 4-1BB co-stimulation would also promote the durability of immune attack, given the distinct and potentially complementary kinetics of proliferation and effector function elicited by 28ζ and BBζ 2G CAR T cells.^45^ To test this, we used a previously described 2G CAR targeted against the pan-ErbB network using the T1E chimeric polypeptide,^42^ here referred to as 2G-T (CD28) (Figure 6A). In pre-clinical testing, we found that regional delivery of 2G-T (CD28) cells elicited disease eradication in a HN3 xenograft model of head and neck squamous cell carcinoma (HNSCC).^42^ This model provided an opportunity to compare durability of CAR and pCAR T cell function in the setting of delayed tumor re-challenge. A panErbB-specific pCAR designated *pCAR-T/T* was engineered by co-expressing a T1E-41BB CCR alongside 2G-T1E CAR (Figure 6A). Equivalent cell surface CAR and CCR co-expression was demonstrated in transduced human T cells by flow cytometry (Figure 6B). T cells engineered to express *pCAR-T/T* exhibited a trend toward enhanced cytotoxicity (Figure 6C) and cytokine release (Figure 6D), when compared with 2G-T (CD28) CAR T cells. *In vivo* activity of 2G-T1E and *pCAR-T/T* T cells was compared in mice with established HN3 HNSCC tumors^42^ (experimental plan [Figure 6E]). Following treatment, bioluminescence imaging (BLI) confirmed that tumor regression occurred in all mice in both active treatment groups, but not in mice that received PBS or UT control T cells (Figure 6F). Tumor-free mice were re-challenged 63 days post CAR T cell infusion with a second injection of HN3 tumor cells. After a transient increase in BLI signal, values reduced to baseline in *pCAR-T/T*-treated mice. By contrast, tumor burden was re-established in 2G-T (CD28) CAR T cell-treated mice (Figure 6G). These data demonstrate the superior functional persistence and long-term anti-tumor responses/immunity mediated by pCAR T cells *in vivo*, when compared with a conventional CAR with equivalent target specificity.

### pCAR T cells demonstrate flexible targeting over a range of relative CAR and CCR affinities

**Figure 7:**
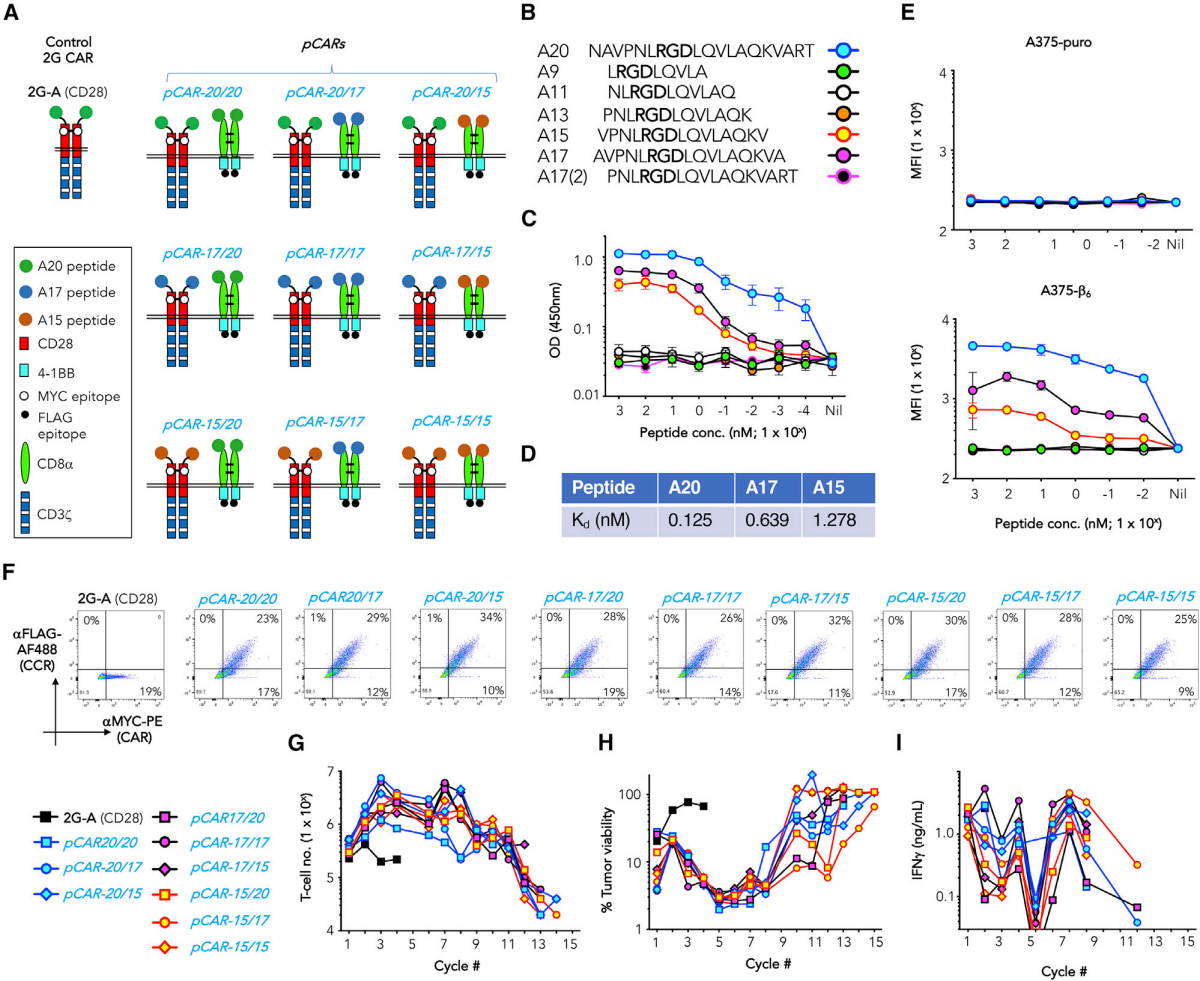
Evaluation of affinity modified pCARs targeted against avb6 integrin (A) Cartoon structures illustrating αvβ6-specific 2G-A (CD28) CAR and derived pCARs. (B) Amino acid sequence of the αvβ6 integrin-binding A20 peptide and truncated derivatives (arginine glycine aspartic acid [RGD] integrin binding motif in bold). (C) Binding of biotinylated A20 and derived truncated peptides to immobilized αvβ6 integrin (mean ± SEM, n = 8). (D) Calculated K_d_ values from binding curves are also shown. (E) Binding of the indicated biotinylated peptides to A375-puro (lack αvβ6 integrin) and A376-b6 (express αvβ6 integrin) was determined by flow cytometry (MFI; mean ± SEM, n = 2). (F) Representative examples of expression of chimeric receptors shown in (A) in permeabilized human T cells. (G) T cells (10^5^) that expressed the indicated CAR or pCAR were co-cultivated with an equal number of BxPC3 pancreatic tumor cells in the presence of IL-2 (100 U/mL, added 2 times per week). Each week, T cells were transferred to a fresh monolayer of 10^5^ BxPC3 tumor cells. T cell number at the end of each stimulation cycle is shown. (H) Tumor cell viability at the end of each stimulation cycle is shown. (I) IFNγ was analyzed in supernatants collected at the end of each stimulation cycle. Data shown in (G) to (I) are representative of three independent replicate experiments.

One potential limiting factor in the successful design of pCARs pertains to the relative affinity of the CAR and CCR for target antigen. To investigate this, a pCAR panel was engineered from a 2G (28ζ) CAR with specificity for αvβ6 integrin, here referred to as 2G-A (CD28) (Figure 7A).^46^ αvβ6 integrin is overexpressed in many solid tumors and is amenable to CAR recognition, using a high-affinity 20-mer viral peptide (A20) (Figure 7B).^46^ End-terminal truncation of A20 yielded two peptides (A17 and A15) (Figure 7B), which bind αvβ6 with progressively lower affinity (i.e., higher K_d_), while further peptide shortening abrogated αvβ6 binding (Figures 7C and 7D). Target specificity of A20, A17, and A15 peptides was confirmed by their selective binding to αvβ6^+^ A375-β6 cells, but not to control A375-puro cells, which lack αvβ6 but express many other integrins (Figure 7E)^46^. We engineered a checkerboard panel of nine pCARs in which these three peptides were used in all possible configurations to direct 2G (28ζ) CAR and (41BB) CCR specificity (Figure 7A). Exploiting distinct embedded epitope tags, 1:1 cell surface expression of each CAR and CCR pairing was demonstrated in transduced T cells by flow cytometry (Figure 7F). When tested in tumor re-stimulation assays using αvβ6^+^ BxPC3 cells, all nine pCARs performed similarly. All exceeded the 2G-A (CD28) CAR to direct sustained T cell expansion (Figure 7G), tumor-cell killing (Figure 7H), and cytokine release (Figure 7I) over multiple stimulation cycles. These data demonstrate that within the sub- to nanomolar range, the pCAR platform is functional across a range of relative CAR/CCR affinities, making it a highly flexible therapeutic option.

## Discussion

A major hurdle in cancer immunotherapy is the onset of tumor-induced T cell dysfunction.^29^^,^^40^^,^^47^ This is primarily driven by sustained antigen over-exposure within a profoundly immunosuppressive TME, aggravated by an imbalance between co-stimulatory and co-inhibitory receptor signaling.^47^ There is considerable evidence that provision of both CD28 and 41BB co-stimulation can synergistically enhance T cell immune responses,^18^, ^19^, ^20^ potentially offering a solution to this important obstacle. Although these receptors activate overlapping signaling pathways, strength and kinetics of response differ markedly. While CD28-containing CARs elicit faster and larger scale signaling flux, 41BB favors a less intense but longer lasting response.^48^

We hypothesized that co-expression of separate chimeric receptors in which both CD28 and 41BB were positioned next to the plasma membrane would provide dual tumor antigen-dependent co-stimulation, thereby mitigating tumor-induced CAR T cell dysfunction. To test this, we developed a single vector-encoded pCAR platform in which a 2G (28ζ) CAR is co-expressed with a 41BB-containing CCR. Delivery of CD28 co-stimulation by the pCAR system results in cytokine release and proliferation,^4^ while provision of 41BB co-stimulation was confirmed by antigen-dependent NF-κB activation.^30^ Using repetitive stimulation assays to model tumor-induced exhaustion,^29^ we found that pCAR T cells with specificity for a single or multiple targets consistently outperformed other dual co-stimulatory systems or 2G CARs that contain either 41BB or CD28 alone. Attenuated tumor-induced dysfunction of pCAR T cells was indicated by enhanced proliferation, cytokine release, and cytolytic function—accompanied by lowered expression of markers of exhaustion and senescence. Flexibility of the system was demonstrated using pCARs that contained a range of targeting moieties (i.e., peptides, cytokine derivatives, and scFvs). While targeting of the same epitope was effective, antigen downregulation could favor immune escape. Nonetheless, we also showed that pCARs can be successfully directed against two or more antigens, with potentiation of activity when both CAR and CCR targets are present. Importantly from a safety perspective, activation of pCAR T cells remained strictly dependent on CAR target engagement. Superior functional persistence of pCAR T cells was indicated by their greater ability to reject tumor re-challenge. The durable nature of anti-tumor immunity mediated by pCAR T cells is a highly attractive attribute for clinical translation.

Optimal T cell responses not only require TCR engagement (signal 1) and co-stimulation (signal 2), but also cytokine support (signal 3). When compared with controls, pCAR T cells demonstrated enhanced and more sustained IL-2 release over repetitive tumor re-stimulation, accompanied by increased gene expression related to JAK-STAT signaling. Maintained ability to release IL-2 is associated with more effective CD8^+^ T cell immune responses in infectious disease models^49^ and the prevention of T cell tolerance within immunosuppressive environments.^50^ Autocrine IL-2 and JAK-STAT signaling supports T cell proliferation and effector function, while also promoting memory formation,^51^ attributes that have enhanced the functionality of CAR T cells.^52^

Co-stimulation by CD28^35^ and 41BB^53^ have each been implicated in the maintenance of T cell metabolic fitness. Exhausted T cells manifest decreased glucose uptake^54^ and defective mitochondrial function.^55^ However, effective dual co-stimulation using the pCAR system resulted in greater upregulation of markers implicated in nutrient uptake, accompanied by superior retention of functional mitochondria.

The consistently superior performance of pCAR T cells highlights a general principle for effective CAR design, namely the importance of juxta-membrane positioning for co-stimulatory signaling. While this has long been established for CD28,^4^ we demonstrate here that 41BB has a similar requirement for optimal function. This provides a rationale for the segregation of distinct co-stimulatory modules within separate synthetic receptors. In keeping with this, we found that the anti-tumor activity of 3G CAR T cells did not exceed that of 2G counterparts, nor did 3G receptors in which 41BB was placed distally elicit the same intensity of NF-κB activation as 2G (BBζ) CARs. A similar explanation is likely to underlie the ability of pCAR T cells to outperform a configuration in which a 1G CAR was co-expressed with a dual CD28+41BB CCR.^25^

Another important property of the pCAR system relates to a signaling-independent potentiating effect of the CCR. This is illustrated by comparison of a MUC1-specific 2G CAR and a control pCAR in which the same MUC1-specific CAR was paired with an endodomain-deficient panErbB-specific CCR. Despite the signaling defective nature of the CCR, these control pCAR T cells achieved greater anti-tumor activity than 2G counterparts. Since the large MUC1 ectodomain sterically hinders T cell engagement,^13^ this increment in function likely reflects a “docking effect” of the CCR to favor closer T cell/ target cell interaction, either alone (e.g., truncated CCR) or in combination with the additional provision of 41BB signaling (intact CCR). This phenomenon may also account for the previously reported functional rescue of a sub-optimal 1G CAR by a co-expressed dual CD28+41BB CCR.^25^ Nonetheless, our data highlight the compromised quality of co-stimulation delivered by a fused CD28 and 41BB endodomain, either within a 3G CAR or a dual CCR. Instead, our findings support the design of chimeric receptor systems in which co-stimulatory units are aligned laterally rather than vertically, thereby achieving membrane proximity.

We also explored the role of affinity in the design of pCARs. Clinical translation of the pCAR approach would be challenging if precise affinity tuning was necessary for every antigen or antigen pair. A panel of αvβ6-specific pCARs was constructed in which CAR affinity exceeded that of the CCR for the same epitope, or vice versa. Across a range of high picomolar to nanomolar K_d_ values, all pCARs behaved in a broadly similar fashion. While this should be confirmed for other antigen(s), these data suggest that the pCAR framework provides flexibility with respect to relative affinity of the CAR and CCR.

While the pCAR platform affords a number of functional advantages over traditional linear CAR fusions, further optimization of the system warrants consideration. Here, we used ribosomal skip peptides to achieve stoichiometric co-expression of both CAR and CCR. However, modification of the ratio of expression of these pCAR constituents could further enhance anti-tumor activity.

It should be noted that CARs and pCARs targeted against M-CSFR were co-expressed with 4αβ, an IL-4-responsive chimeric cytokine receptor.^27^ However, we feel that this is unlikely to have influenced our results since it was a consistent variable in all M-CSFR-targeted T cell populations and was not used in the other models presented here in which superiority of pCAR function was also shown. T cells that express 4αβ have potent *in vivo* anti-tumor activity that is comparable to that seen with CAR alone.^42^^,^^43^

In summary, we describe a CAR technology that effectively delivers integrated co-stimulation by CD28 and 41BB, provided that co-stimulatory modules are located in their natural membrane-associated position. Our data suggest that pCAR signaling promotes differentiation into long-lived memory T cells *in vivo*, allowing enhanced persistence, function, and long-term protection of the recipient. Together, these data support the development of pCAR-based immunotherapy for tumor types that have proven resistant to therapeutic intervention.

### Limitations of this study

*In vivo* studies did not contain a uniform panel of control CARs owing to the very large number of constructs generated in this study. Another limitation pertains to the lack of *in vivo* testing of pCARs with altered affinity. Finally, our article only describes the exemplification of pCARs that contain CD28 and 41BB. Future investigation of other combinations of co-stimulatory modules and mutated derivatives is warranted.

## STAR★Methods

### Key resources table

**Table:** 

REAGENT or RESOURCE	SOURCE	IDENTIFIER
**Antibodies**		

Anti-c-Myc (9e10) hybridoma supernatant	ECACC (hybridoma)	N/A
Anti-Flag tag - PE (L5)	BioLegend	Cat# 637310, RRID:AB_2563148
Anti-goat Ig FITC-conjugated (rabbit polyclonal)	DAKO (Agilent)	Cat# F0250
Anti-GLUT1 - Alexa Fluor 647 (EPR3915)	Abcam	Cat# ab195020, RRID:AB_2783877
Anti-human CD3 - APC/Cy7	BioLegend	Cat# 344818, RRID:AB_10645474
Anti-human CD8 - PE-Cy7 (SK1)	BioLegend	Cat# 344711, RRID:AB_2044007
Anti-human CD4 - PE/Cy7 (RPA-T4)	BioLegend	Cat# 300512, RRID:AB_314080
Anti-human CD45RA - V450 (HI100)	BD Biosciences	Cat# 560362, RRID:AB_1645575
Anti-human CD45RO - PerCP/Cy5.5 (UCHL1)	BioLegend	Cat# 304221, RRID:AB_1575041
Anti-human CD57 - APC (QA17A04)	BioLegend	Cat# 393305, RRID:AB_2734459
Anti-human CD69 - BV605 (FN50)	BioLegend	Cat# 310938; RRID:AB_2562306
Anti-human CD71 -PerCP Cy5.5	BioLegend	Cat# 334114; RRID:AB_2563175
Anti-human CD98 - PE-Vio770	Miltenyi Biotec	Cat# 130-105-710, RRID:AB_2659686
Anti-human CD115 - CSF1R (3-4A4)	Santa-Cruz	Cat# sc-02, RRID: AB_627624
Anti-human CD124 - PE (hIL4R-M57)	BD Biosciences	Cat# 552178, RRID:AB_394355
Anti-human CD197 (CCR7) - APC (G043H7)	BioLegend	Cat# 353213, RRID:AB_353213
Anti-human CD247 (CD3z) - 1D4	BD Biosciences	Cat# 556366, RRID:AB_396389
Anti-human CD279 (PD1) - APC/Cy7 (EH12.2H7)	BioLegend	Cat# 329921, RRID:AB_10900982
Anti-human CD279 (PD1) - PE/Dazzle 594 (EH12.2H7)	BioLegend	Cat# 329939, RRID:AB_2563658
Anti-human CD366 (Tim-3) - APC	Biolegend	Cat# 345011, RRID:AB_2561717
Anti-human CSF1 (goat polyclonal)	Sigma-Aldrich	Cat# M9182, RRID:AB_260717
Anti-human CSF1 (monoclonal)	BioLegend	Cat# 699203, RRID:AB_2715880
Anti-human EGF (10825)	R&D Systems	Cat# MAB236
Anti-human EGF - biotin (polyclonal)	R&D Systems	Cat# BAF236
Anti-human KLRG1 PE-Vio 615	Miltenyi Biotec	Cat# 130-120-427, RRID:AB_2784407
Anti-human IgG Fc - APC (HP6017)	BioLegend	Cat# 409305, RRID:AB_11150590
Anti-human IL-34 - biotin (E033B8)	BioLegend	Cat# 361402, RRID:AB_2563035
Anti-human IL-34 (1D12)	Abcam	Cat# ab101443, RRID:AB_10711208
Anti-mouse IgG - APC (goat polyclonal)	BioLegend	Cat# 405308, RRID:AB_315011
Anti-mouse IgG - PE (goat polyclonal)	ThermoFisher Scientific	Cat# M30004-4, RRID:AB_2536618
Anti-rat IgG - FITC (goat polyclonal)	BioLegend	Cat# 405404, RRID:AB_315015
Anti-rat IgG - PE (goat polyclonal)	ThermoFisher Scientific	Cat# A10545, RRID:AB_1500703
Anti-rat IgG - APC (Poly4054; goat polyclonal)	BioLegend	Cat# 405407, RRID:AB_315018
Isotype control antibodies	ThermoFisher Scientific	N/A

**Chemicals, peptides, and recombinant proteins**		

Brefeldin A solution	BioLegend	Cat# 420601
CellTrace™ CFSE Cell Proliferation Kit	ThermoFisher Scientific	Cat# C34554
D-luciferin	R&D Systems (Biotechne)	Cat# 122799
DAPI staining solution	Miltenyi Biotec	Cat# 130-111-570
Dynabeads™ human T-activator CD3/CD28	ThermoFisher Scientific	Cat# 11132D
EGFR Fc chimera	R&D Systems (Biotechne)	Cat# 344-ER
ErbB2/HER2 Fc chimera	R&D Systems (Biotechne)	Cat# 1129-ER
GeneJuice transfection reagent	Merck Chemicals Ltd	Cat# 70967-4
Live/Dead Fixable dead cell stain, blue	ThermoFisher Scientific	Cat# L23105
MTT (3-(4,5-Dimethylthiazol-2-yl)-2,5-diphenyl-2H-tetrazolium bromide)	Apollo Scientific	Cat# BID2165
MUC1 60-mer - biotin (VTSAPDTRPAPGSTAPPAHG)3	NeoMPS; Wilkie et al., 2008^13^	PMID: 18354214
Phusion® High-Fidelity PCR Master Mix with HF Buffer	New England BioLabs	Cat# M0531
Phytohemagglutinin-L	Sigma-Aldrich	Cat# 11249738001
Recombinant human IL-2	Peprotech EC	Cat# 200-02
Recombinant human IL-4	Peprotech EC	Cat# 200-04
RetroNectin	Takara	Cat# 60690
Streptavidin APC	BioLegend	Cat# 405207
Streptavidin PE	BioLegend	Cat# 405204
Streptavidin PE/Cy5	BioLegend	Cat# 405205
Tetramethylrhodamine, ethyl ester	ThermoFisher	Cat# T669

**Critical commercial assays**		

IFN-γ ELISA kit	ThermoFisher Scientific	Cat# 88-7316-76, RRID:AB_2575072
IL-2 ELISA kit	ThermoFisher Scientific	Cat# 88-7025-76, RRID:AB_2574956
IL-34 Duoset ELISA kit	R&D Systems (Biotechne)	Cat# DY5265B
M-CSF Duoset ELISA kit	R&D Systems (Biotechne)	Cat# DY216
nCounter Sprint Cartridge SPRINT-CAR-1.0	NanoString Technologies Inc	Cat# 100078
One-Step™ Luciferase Assay System	BPS Bioscience	Cat# 60690-1
PlasmoTest™	InvivoGen	Cat# rep-pt1
RNeasy® Mini Kit	QIAGEN	Cat# 74104
Qubit™ RNA BR Assay Kit	ThermoFisher Scientific	Cat# Q10210
XT_HS_CAR-T Panel_CSO XT-CSO-HCART1-12 (CAR-T Characterization Panel)	NanoString Technologies Inc	Cat# 115000343
		N/A

**Deposited data**		

NanoString gene expression data	This paper	GEO: GSE186557

**Experimental models: Cell lines**		

293VEC-RD114™	Dr Manuel Caruso, Centre de recherche du CHU de Québec, Canada	https://www.biovecpharma.com/products.php?id=19
K299 (Karpas-299)	Dr Stephan Mathas, Max-Delbrück-Center for Molecular Medicine, Germany	DSMZ Cat# ACC-31, RRID:CVCL_1324
MDA-MB-468	Breast Cancer Now Research Unit, King’s College London	NCI-DTP Cat# MDA-MB-468, RRID:CVCL_0419
MDA-MB-435	Breast Cancer Biology Group, King’s College London	NCI-DTP Cat# MDA-MB-435, RRID:CVCL_0417
MDA-MB-435 MUC1+ HER2+	This lab; Wilkie et al., 2008^13^	N/A
NF-κB luciferase reporter Jurkat	BPS Bioscience (distributed by Tebu-Bio)	Cat# 60651
PG13	European Collection of Cell Cultures (ECACC)	ATCC Cat# CRL-10685, RRID:CVCL_8933
T47D	Breast Cancer Now Research Unit, King’s College London	NCI-DTP Cat# T-47D, RRID:CVCL_0553
T47D *FMS*	This manuscript	N/A

**Experimental models: Organisms/strains**		

Mouse: NSG® (NOD.Cg-Prkdc^scid^ Il2rg^tm1Wjl^/SzJ)	Charles River	Strain code: 614
Mouse: Fox Chase SCID Beige (CB17.Cg-Prkdc^scid^Lyst^bg-J^/Crl)	Charles River	Strain code: 250

**Oligonucleotides**		

Primers for PIPE cloning	See Table S1	N/A

**Recombinant DNA**		

2G-M (CD28)	This manuscript	N/A
2G-M (41BB)	This manuscript	N/A
3G-M	This manuscript	N/A
Trunc.-M	This manuscript	N/A
2G-34 (CD28)	This manuscript	N/A
3G-34	This manuscript	N/A
Trunc.-34	This manuscript	N/A
*pCAR-M/34*	This manuscript	N/A
Trunc. CAR in pCAR	This manuscript	N/A
1G CAR/ Dual CCR	This manuscript	N/A
2G-H (CD28)	This lab; Wilkie et al., 2008^13^	PMID: 18354214
2G-H (41BB)	This manuscript	N/A
3G-H	This manuscript	N/A
*pCAR-H/T*	This manuscript	N/A
2G-T (CD28) (previously referred to as T1E28z)	This lab; Davies et al., 2012^42^	PMID: 22354215
*pCAR-T/T*	This manuscript	N/A
A-2 (previously referred to as A20-28z)	This lab; Whilding et al., 2017^46^	PMID: 28129120
*pCAR-20/20*	This manuscript	N/A
*pCAR-20/17*	This manuscript	N/A
*pCAR-20/15*	This manuscript	N/A
*pCAR-17/20*	This manuscript	N/A
*pCAR-17/17*	This manuscript	N/A
*pCAR-17/15*	This manuscript	N/A
*pCAR-15/20*	This manuscript	N/A
*pCAR-15/17*	This manuscript	N/A
*pCAR-15/15*	This manuscript	N/A
*c-fms* (*CSF1R* gene)	This lab; Lo et al., 2008^56^	PMID: 17950877
HER2	This lab; Wilkie et al., 2012^24^	PMID: 22526592
RFP/ffLuc	This lab; Lamprecht et al., 2010^57^	PMID: 20436485
Human CSF-1 isoform 3	Gift of Dr. Kirsten Koths, Chiron Corporation	Uniprot P09603-3
T1E peptide	This lab; Davies et al., 2012^42^	PMID: 22354215
ICR12	This lab; Wilkie et al., 2012^24^	PMID: 22526592
FMC63 scFv	Kochenderfer et al., 2009^58^	GenBank: HM852952.1
4αβ	Wilkie et al., 2010^27^	PMID: 20562098
RD114	Gift of Prof. M. Collins, University College London	N/A
pEQ-Pam3	Gift of Dr. M. Pulé, University College London	N/A

**Software and algorithms**		

CellQuest Pro	BD Biosciences	N/A
FACSDiva	BD Biosciences	N/A
FlowJo	FlowJo, LCC, BD Biosciences	N/A
MARS Data Analysis Software	BMG Labtech	N/A
BMG Labtech Control Software	BMG Labtech	N/A
GraphPad Prism version 5.0, 6.0 and 7.0	GraphPad software	N/A
Excel for Mac 2011	Microsoft	N/A
Living Image Software	PerkinElmer	N/A
R, package NanoStringNorm	Waggot et al., 2012^60^	PMID: 22513995
ClustVis	Metsalu et al., 2015^61^	PMID: 25969447
GSEA v4.0.3 for Windows	Broad Institute; Subramanian et al., 2005^62^	PMID: 16199517

### Resource availability

#### Lead contact

Further information and requests for resources and reagents should be directed to and will be fulfilled by the Lead Contact, John Maher (john.maher@kcl.ac.uk).

#### Materials availability

Reagents generated in this study will be made available on request, but we may require a payment and/or a completed Materials Transfer Agreement if there is potential for commercial application.

### Experimental model and subject details

#### Mice

SCID Beige and Nod SCID γc^null^ (NSG) mice were housed in Biological Services Units at King’s College London.

#### Cell Lines and Tissue Culture

Cell lines and their origin are listed in the Key resources table. Tumor cell lines were grown in R10 or D10 medium, respectively comprising RPMI or DMEM supplemented with 10% FBS and GlutaMax. PG13 and 293VEC-RD114 cells retroviral packaging cells were maintained in D10. Cells were maintained at 37°C in a humidified atmosphere of 5% CO_2_. Cell lines were validated by STR typing and were routinely monitored for mycoplasma contamination. Where indicated, cell lines were engineered to express RFP/ffLuc,^46^ human *FMS*,^56^ (*M-CSFR* gene), HER2,^24^ or MUC1/HER2.^13^

#### Human Study Oversight

Blood samples were obtained from healthy male and female volunteers aged between 18-65 years old with approval of a National Health Service Research Ethics Committee (reference 09/H0804/92 and 18/WS/0047).

### Method details

#### Retroviral Constructs

The 2G-M (CD28) and Trunc.-M constructs were generated by fusion of a human CD8α leader to codons 33-189 of human M-CSF isoform 3 (Uniprot P09603-3) by overlap extension PCR using primers listed in Table S1. The resultant NcoI/NotI flanked fragment was inserted into plasmids dubbed SFG T1E28z or SFG T1NA in place of the existing NcoI/ NotI fragment.^42^ M-CSFR-targeting *pCAR-M/34*, 3G-M, 2G-34, Trunc.-34 and 3G-34 constructs were constructed using the Polymerase Incomplete Primer Extension (PIPE) cloning method using primers listed in Table S1. The IL-34 sequence (isoform 1) encoded codons 1-242. The 2G-H (CD28)^13^ and 2G-A (CD28) CARs^46^ and T1E peptide^42^ have been previously described. The 2G-T (CD28) CAR was generated by mutagenesis of the previously described T1E-28z CAR,^42^ replacing the MYPPPY motif in the CD28 extracellular domain with a EQKLISEEDL 9e10 epitope tag (Mr Gene, Regensburg, Germany). All other CARs and pCARs were constructed by gene synthesis and cloning (Genscript, Hong Kong, China and Leiden, the Netherlands) using human codon optimized sequences and ligation of digested DNA fragments as appropriate. In all cases, the CD28 sequence incorporated codons 114-220 or, where truncated (Trunc.) to inactivate signaling, codons 114-182. In all cases, the CD8α spacer and transmembrane sequence incorporated codons 137-208 while the 41BB endodomain sequence encoded for codons 214-255. All M-CSF-targeted CARs and pCARs were co-expressed in human T cells with 4αβ, a chimeric cytokine receptor that provides a selective IL2/15 signal upon binding of IL4.^27^ Stoichiometric co-expression of two or three transgenes was achieved using one or more intervening furin cleavage sites (RRKR) followed by a [serine-glycine]_2_ linker and either a *Thosea Asigna* 2A (T2A) or *Porcine Teschovirus* (P2A) ribosomal skip peptide (codon wobbled where necessary). To visualize tumors *in vivo*, ffLuc was co-expressed with RFP as described.^46^ The SFG encoded M-CSFR^56^ and HER2^24^ constructs were previously described.

#### Transduction and Expansion of Human T Cells

Viral vector was prepared as described using PG13 cell lines, 293VEC-RD114 cells or by triple transfection of 293T cells. In brief, 1.65x10^6^ low passage 293T cells in 11mL IMDM + 10% FBS were evenly distributed in a 10cm plate. After 8-24h, GeneJuice (30μL) was added to 470μL IMDM (no serum) and mixed gently. After incubation for 5 min at room temperature, 3.125μg RD114 plasmid, 4.6875μg pEQ-Pam3 plasmid and 4.6875μg SFG vector of interest were added to the GeneJuice/medium mixture, mixed gently and incubated for 15 min at room temperature. The transfection mixture was dropwise to the plate and gently swirled to ensure even distribution. After incubation for 48h at 37°C, 5% CO_2,_ medium was removed for snap freezing using an ethanol dry ice bath and replaced. After a further 24h, this procedure was repeated. Frozen virus was stored in aliquots at −80°C. Retroviral transduction and culture of phytohemagglutinin- or CD3+CD28 Dynabead-activated T cells using RetroNectin-coated plasticware was performed as described.^4^^,^^59^

#### Flow Cytometry Analysis

All cell staining reactions were performed on ice. For intracellular antigen detection, cells were stained with a fixable Live/Dead dye before being stained for surface proteins for 30 min on ice. Intracellular staining was performed by fixation with 0.01% formaldehyde followed by permeabilization using PBS + 0.5% BSA + 0.1% saponin. Cells were subsequently stained for intracellular proteins for 30 min at 4°C. All gates were set using isotype control antibodies or fluorescence minus one controls. Where necessary, a viability stain was included and non-specific binding of the antibodies was limited by using an appropriate Fc blocking reagent prior to the staining steps. Flow cytometry was performed using a FACSCalibur cytometer with CellQuest Pro software or BD LSRFortessa cytometer with BD FACSDiva software and data was analyzed using FlowJo, LLC.

#### Enzyme-linked Immunosorbent Assay

Supernatants collected from co-culture of tumor cells with CAR T cells were analyzed using a human IFNγ or human IL2 enzyme-linked immunosorbent assay (ELISA) as described by the manufacturers. In pooled re-stimulation assays, cytokine production was set to zero in each cycle after T cell cultures failed.

#### Cytotoxicity Assays

Tumor cells were incubated with T cells at specified effector to target (E:T) ratios. In the case of adherent targets, residual tumor cell viability was quantified using an MTT assay. After removal of the supernatant and residual T cells, MTT was added at 500 μg/mL in D10 medium for 40 min at 37°C and 5% CO_2_. Formazan crystals were resuspended in DMSO and absorbance was measured at 560 nm. Alternatively, tumor cell viability was monitored by luciferase assays. D-luciferin was added at 150 mg/mL immediately prior to luminescence reading. In each case, tumor cell viability was calculated as follows: (absorbance or luminescence of tumor cells cultured with T cells/absorbance or luminescence of untreated monolayer alone) x 100%. In pooled re-stimulation assays, tumor viability was set to 100% in each cycle after T cell cultures failed.

#### Tumor Re-stimulation Assays

Transduced T cells were co-cultured with tumor cell lines in the absence of exogenous cytokine support. In the case of M-CSFR-targeted T cells, 1 × 10^6^ T cells were cultured on a confluent tumor monolayer (24 well plate). In other cases, CAR T cells were added to tumor cells at numbers specified in individual experiments. Once or twice per week, T cells were recovered and re-stimulated on new tumor cells. Supernatant was harvested after 24h for cytokine analysis while tumor cell viability was determined after 24h or 72h by MTT or luciferase assay. If T cells could not be re-stimulated, tumor viability was set to 100% and cytokine production was set to zero.

#### NFκB Reporter Assay

Jurkat cells that express an NFκB reporter were engineered to express the indicated CARs by retroviral transduction. 0.2 × 10^6^ cells were incubated on confluent T47D *FMS* monolayers in 100 μL D10 medium in a 96-well plate for 5h, after which 100 μL One-Step Luciferase Agent was added. The plate was gently rocked for 20 min at room temperature, protected from light, to allow full cell lysis, after which luminescence was measured.

#### Mitochondrial Membrane Potential

T cells were incubated for 30 min at 37°C with 100 nM Tetramethylrhodamine, ethyl ester (TMRE) in RPMI, after which they were washed with RPMI twice before being incubated with antibodies for cell surface staining without fixation.

#### RNA Analysis

M-CSFR-targeting CAR T cells were labeled using the CellTrace™ CFSE Cell Proliferation Kit according to the manufacturer’s protocol using a final concentration of 10 mM CFSE. Labeled cells (1 × 10^6^ cells) were stimulated on a confluent T47D or T47D *FMS* monolayer (24 well plate). After 24h, live (DAPI^-^), CFSE^+^ T cells were flow sorted on a BD FACSAria™ II Cell Sorter with BD FACSDiva Software and whole cell lysates were produced by syringe homogenization of 0.5-2.6 × 10^6^ cells in 350 μL Buffer RLT. RNA was isolated using the RNeasy® Mini Kit according to the manufacturer’s protocol. RNA purity was determined using a NanoDrop Spectrophotometer and RNA quantity was determined using a Qubit Fluorometer and Qubit RNA BR Assay Kit. All samples had a total RNA concentration greater than 23ng/μL and were, therefore, included in the analysis. Eighty ng RNA was used for gene expression analysis using the CAR-T Characterization Panel and NanoString nCounter® Sprint Profiler following the manufacturer’s instructions.

#### *In vivo* Xenograft Studies

All *in vivo* experimentation adhered to UK Home Office guidelines, as specified in project license number 70/7794 or P23115EBF and was approved by the King’s College London animal welfare and ethical review body (AWERB). Mice were purchased from Charles River Laboratories and were 6-10 weeks old when used for experiments. Female mice were used for all breast cancer studies, and male mice for all other xenograft studies. Mice were allocated to experimental groups based on similar average tumor burden prior to treatment.

Where indicated, tumor cells were transduced with SFG ffLuc/RFP and were purified by flow sorting prior to engraftment *in vivo*. Intraperitoneal tumor models were established by inoculation of MDA-MB-468, HER2-engineered MDA-MB-468 or HN3 cells (1 × 10^6^ cells each). K299 tumor cells were injected i.v. at 2 × 10^6^ cells.

Engineered T cells were administered i.p. (MDA-MB-468, HN3) or i.v. (K299) as specified in individual experiments. Bioluminescence imaging was performed using an IVIS Spectrum Imaging platform (PerkinElmer) with Living Image software. To monitor tumor status, mice were injected i.p. with D-luciferin (150 mg/kg) and imaged under isoflurane anesthesia after 20 min. In all experiments, animals were inspected daily and weighed weekly.

#### Gene Expression Analysis

For RNA expression analysis, raw data (RCC files) were received from NanoString and used directly as input for the open source R package, NanoStringNorm^60^ for background correction and between-sample normalization. Principal Component Analysis (PCA) was performed using ClustVis.^61^ Unit variance scaling was applied to rows and singular value decomposition with imputation was used to calculate principal components. Prediction ellipses were such that with probability 0.95, a new observation from the same group would fall inside the ellipse. Gene set enrichment analysis (GSEA) was performed on canonical pathways curated gene sets from the Broad Molecular Signature Database (https://www.broadinstitute.org/gsea/msigdb/) using the gene_set permutation type.^62^

### Quantification and statistical analysis

#### Statistical Analysis

All data are derived from biological replicates involving independent donors unless otherwise indicated. For analysis of multiple groups, statistical analysis was performed using one-way or two-way ANOVA test (depending on the number of independent variables) followed by Tukey’s multiple comparisons test. For non-parametrically distributed data, a Kruskal Wallis test was performed. Survival data were analyzed using a Log rank (Mantel-Cox) test. When only 2 groups were compared, a Student’s t test or Mann-Whitney test was performed, depending on normality of the data. All statistical analyses were performed using GraphPad Prism version 9.1.

## Data Availability

NanoString data were deposited at NIH Gene Expression Omnibus (GEO) and are publicly available as of the date of publication. There was no new code developed as part of this study. Any additional information required to re-analyze the data reported in this work paper is available from the Lead Contact upon request.

## Supplemental information

Document S1. Figures S1–S4 and Table S1

Document S2. Article plus supplemental information
