# Supplementary material for: Synergistic T cell signaling by 41BB and CD28 is optimally achieved by membrane proximal positioning within parallel chimeric antigen receptors
Source: Cell Rep Med. 2021 Dec 21;2(12):100457. doi: 10.1016/j.xcrm.2021.100457 (PMC8714859; doi:10.1016/j.xcrm.2021.100457)
Supplement: Document S1. Figures S1–S4 and Table S1 [file mmc1.pdf]

Cell Reports Medicine, Volume *2*

Supplemental information

Synergistic T cell signaling by 41BB and CD28 is optimally achieved by membrane proximal positioning within parallel chimeric antigen receptors

Tamara Muliaditan, Leena Halim, Lynsey M. Whilding, Benjamin Draper, Daniela Y. Achkova, Fahima Kausar, Maya Glover, Natasha Bechman, Appitha Arulappu, Jenifer Sanchez, Katie R. Flaherty, Jana Obajdin, Kristiana Grigoriadis, Pierre Antoine, Daniel Larcombe-Young, Caroline M. Hull, Richard Buus, Peter Gordon, Anita Grigoriadis, David M. Davies, Anna Schurich, and John Maher

Supplemental Information

Synergistic signaling by 41BB and CD28 in therapeutic T-cells is optimally achieved by membrane proximal positioning within parallel chimeric antigen receptors

Tamara Muliaditan, Leena Halim, Lynsey M. Whilding, Benjamin Draper, Daniela Y. Achkova, Fahima Kausar, Maya Glover, Natasha Bechman, Appitha Arulappu, Jenifer Sanchez, Katie R. Flaherty, Jana Obajdin, Kristiana Grigoriadis, Pierre Antoine, Daniel Larcombe-Young, Caroline M. Hull, Richard Buus, Peter Gordon, Anita Grigoriadis, David M. Davies, Anna Schurich, John Maher

Table S1. Primers for PIPE cloning *(Related to Figure 1)*

**2G-M** (CD28) **and Trunc.-M**	Fw1	5’-CCACCTCTGACTTGAGCGTCGATTTTTGTGATGCTCGTCA-3’
	Rv1	5’-CCATGGCAGTCTAGAGGATGGTCCACCCCCGGGGTCGGCA-3’
	Fw2	5’-CCATGGCTCTCCCAGTGACTGCCCTACTGCTTCCCCTAGC-3’
	Rv2	5’-GACGCTCAAGTCAGAGGTGGCGAAACCCGACAGGACTATA-3’
	Fw3	5’-CATCCTCTAGACTGCCATGGGCTGGCTGTGTTCCGGCCTG-3’
	Rv3	5’-AGTCACTGGGAGAGCCATGGGTCCGGGGTTCTCTTCCACG-3’
pCAR-M/34	Fw1	5’-TGCAGGCCCTGCCCCCTCGCAGGAGGAAGAGAAGTGGATC-3’
	Rv1	5’-GATCCCTCGAGTGGCTGTTATTACAGCTCGCAGCCGCCCT-3’
	Fw2	5’-TAACAGCCACTCGAGGGATCCGGATTAGTCCAATTTGTTA-3’
	Rv2	5’-TCTGACGCTCAGTGGAACGAAAACTCACGTTAAGGGATTT-3’
	Fw3	5’-TCGTTCCACTGAGCGTCAGACCCCGTAGAAAAGATCAAAG-3’
	Rv3	5’-GAGCCTGTAAGTGAGCTTGGAGAGAGGGGCTGTTAGTAAC-3’
	Fw4	5’-CCAAGCTCACTTACAGGCTCTCTACTTAGTCCAGCACGAA-3’
	Rv4	5’-GCGAGGGGGCAGGGCCTGCATGTGAAGGGCGTCGTAGGTG-3’
**3G-M**	Fw1	5’-AGAGTGAAGTTCAGCAGGAGCGCAGAGCCCCCCGCGTACC-3’
	Rv1	5’-AGCTTTGGTAACAGGAATATTGCAGCATTCATGCACATTG-3’
	Fw2	5’-CAATGTGCATGAATGCTGCAATATTCCTGTTACCAAAGCT-3’
	Rv2	5’-GCGGCCGCTCCCTCGGACTGCCTCTCATGGCCTTGGCTGG-3’
	Fw3	5’-CAGTCCGAGGGAGCGGCCGCAATTGAAGTTATGTATCCTC-3’
	Rv3	5’-CTCCTGCTGAACTTCACTCTCAGCTCGCAGCCGCCCTCCT-3’
**2G-34** (CD28) **and Trunc.-34**	Fw1	5’-AGAGTGAAGTTCAGCAGGAGCGCAGAGCCCCCCGCGTACC-3’
	Rv1	5’-AGCTTTGGTAACAGGAATATTGCAGCATTCATGCACATTG-3’
	Fw2	5’-CAATGTGCATGAATGCTGCAATATTCCTGTTACCAAAGCT-3’
	Rv2	5’-GCGGCCGCTCCCTCGGACTGCCTCTCATGGCCTTGGCTGG-3’
	Fw3	5’-CAGTCCGAGGGAGCGGCCGCAATTGAAGTTATGTATCCTC-3’
	Rv3	5’-CTCCTGCTGAACTTCACTCTCAGCTCGCAGCCGCCCTCCT-3’
**3G-34**	Fw1	5’-AATTGAAGTTATGTATCCTCCTCCTTACCTAGACAATGAG-3’
	Rv1	5’-ATCAGCTCACTCAAAGGCGGTAATACGGTTATCCACAGAA-3’
	Fw2	5’-CCGCCTTTGAGTGAGCTGATACCGCTCGCCGCAGCCGAAC-3’
	Rv2	5’-GGGTCCGGGGTTCTCTTCCACGTCGCCGCAGGTCAGCAGG-3’
	Fw3	5’-TGGAAGAGAACCCCGGACCCATGCCTAGAGGCTTCACATG-3’
	Rv3	5’-GAGGATACATAACTTCAATTGCGGCCGCAGGCAGCAGTCC-3’

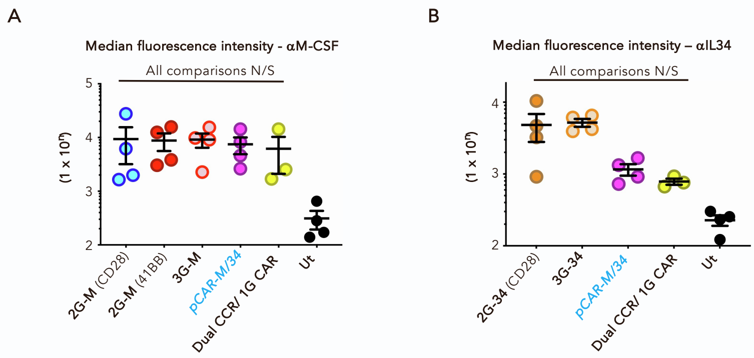

Figure S1. Expression of M-CSFR-targeted chimeric receptors. (A-B) T-cells were engineered to express the indicated CARs or pCAR together with the 4αβ chimeric cytokine receptor and were then enriched by culture in IL4 (n=3-4 independent donors). (A) Expression of the M-CSF-containing chimeric receptor components were quantified by flow cytometry and are expressed as median fluorescence intensity, making comparison with untransduced (Ut) T-cells from the same donor. (B) Expression of the IL34-containing chimeric receptor components were quantified by flow cytometry and are expressed as median fluorescence intensity, making comparison with untransduced (Ut) T-cells from the same donor. Statistical analysis was performed using one-way ANOVA. N/S – not significant. *Related to Figure 1.*

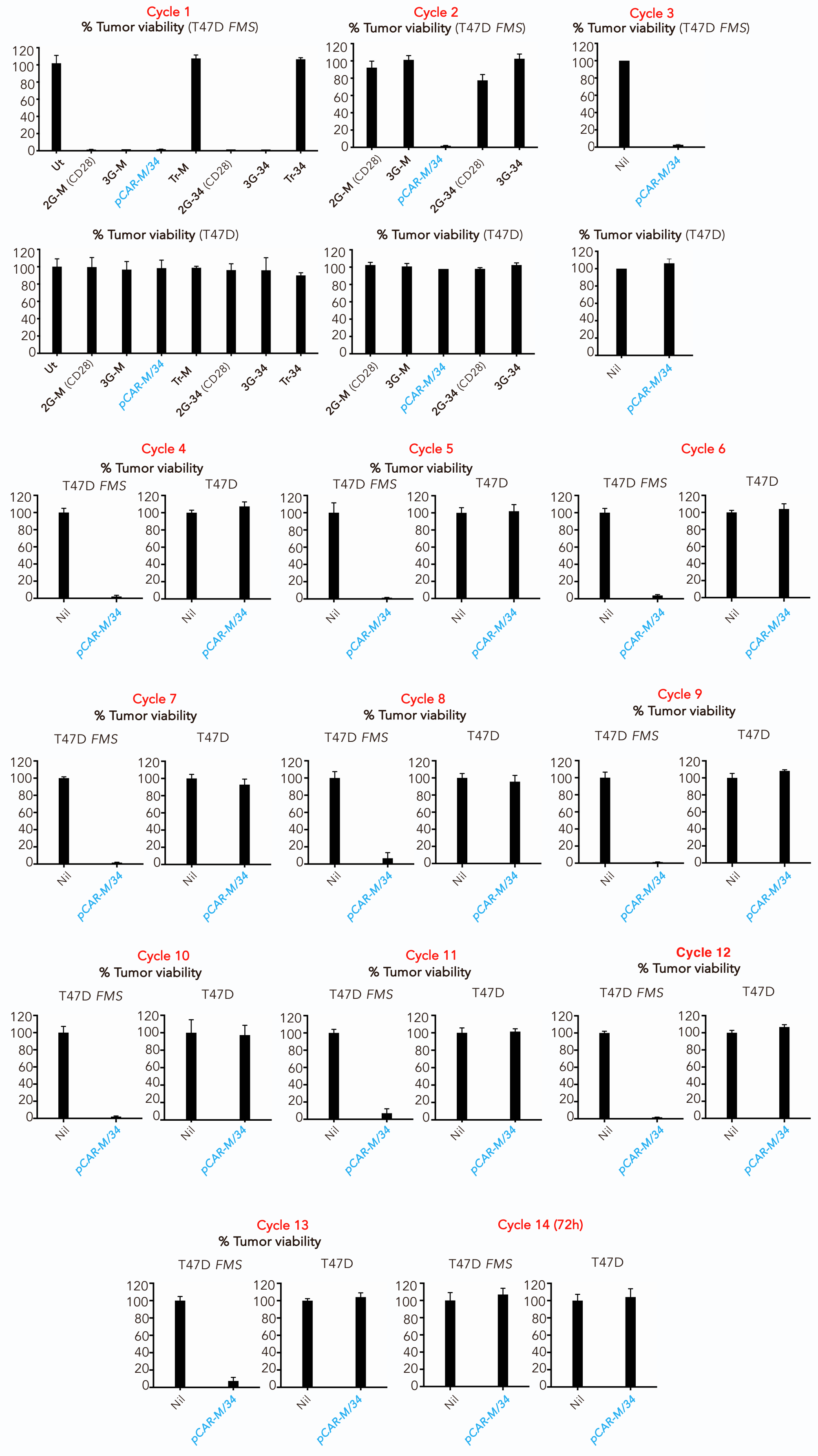

Figure S2. Cytotoxic activity of M-CSFR re-targeted T-cells upon iterative re-stimulation. Representative example in which a panel of M-CSFR-targeted CAR or pCAR T-cells were re-stimulated weekly without cytokine support on T47D *FMS* or T47D tumor cells (1:1 ratio). Tumor cell viability was determined by MTT assay after overnight co-culture (mean ± SD, n=3 technical replicates). Note extended duration of cycle 14 assay (assessed after 72h), which demonstrates that cytotoxic function had been lost in that stimulation cycle. Data are representative of 20 experiments using T-cells from independent donors. *Related to Figure 1.*

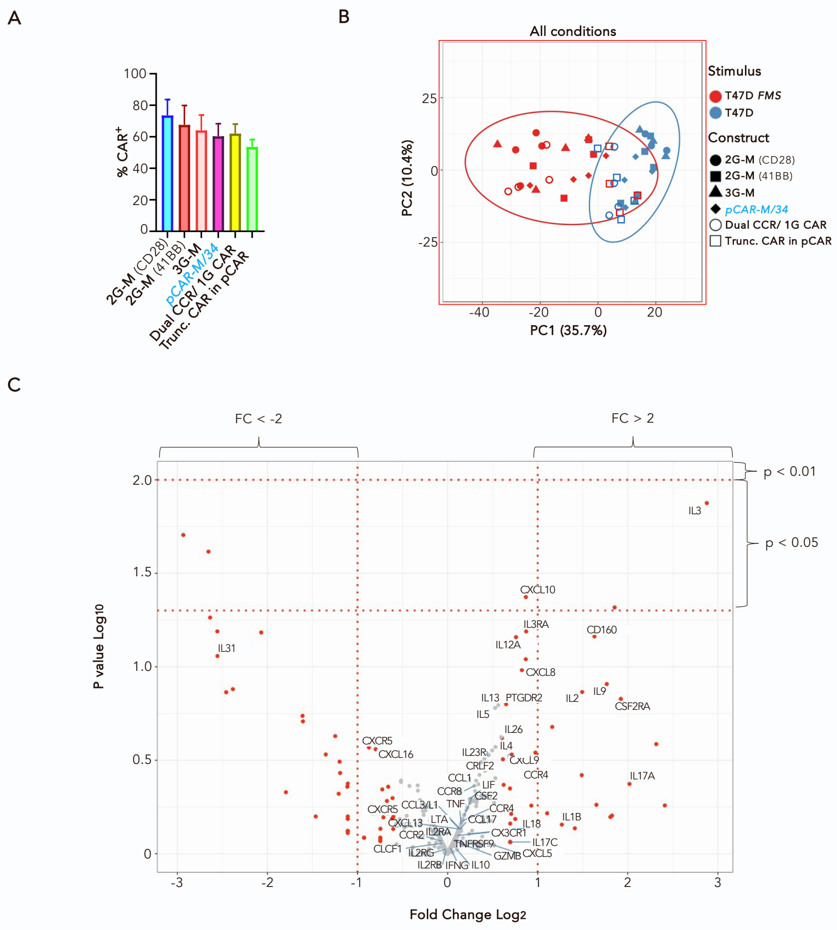

Figure S3. NanoString analysis of M-CSFR re-targeted T-cells. (A) Expression of M-CSFR-specific pCAR and CARs in human T-cells which were used for NanoString gene expression analysis (mean ± SEM, n=4). (B) CFSE-labeled M-CSFR-specific CAR and pCAR T-cells were stimulated for 24h on T47D or T47D *FMS* tumor monolayers and then flow sorted prior to RNA extraction. Principal component analysis indicated that differential gene expression was most evident when comparing stimulated CD3ζ-containing constructs to unstimulated or signaling defective controls. Image was generated using ClustVis with manual superimposition of hollow symbols to overcome inadequate font size. (C) Volcano plot indicating differential gene expression when comparing stimulated *pCAR-M/34* T-cells with all other stimulated M-CSFR-specific CARs (2G-M (CD28), 2G-M (41BB), 3G-M and Dual CCR/ 1G CAR). Cytokine-related genes are highlighted while those genes with fold change (FC) of ≥ 1.5 are indicated in red. *Related to Figure 2.*

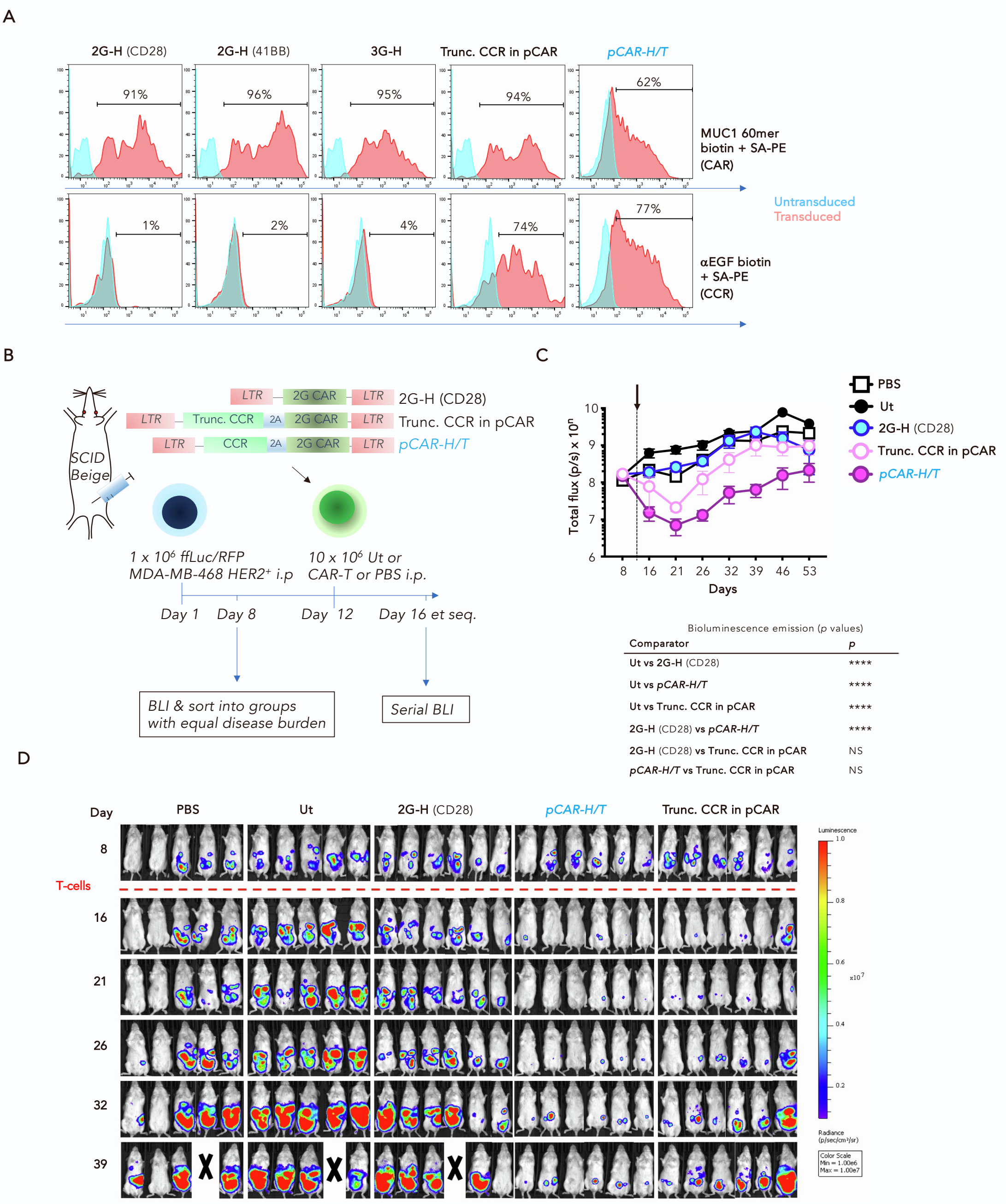

Figure S4. Characterization of MUC1/panErbB-targeted parallel CAR T-cells. (A) Representative examples of T-cell expression of the indicated 2G and 3G CARs, pCAR and truncated pCAR control. Data are representative of 5 or more replicates. (B) Assessment of anti-tumor activity of *pCAR-H/T* pCAR and control T-cells in a model of MUC1/ HER2$^+$ breast cancer. MDA-MB-468 tumor cells naturally express MUC1, EGF receptor and HER3 and were engineered to over-express HER2 by retroviral transduction. SCID Beige mice were inoculated i.p. with 1 x 10^6 ffLuc/RFP$^+$ HER2$^+$ MDA-MB-468 tumor cells. After 12 days, 10 x 10^6 T-cells that express the indicated CAR or pCAR were injected i.p. (arrowed). (C) Tumor burden in mice treated as described in panel B was monitored using BLI (mean ± SEM, n=5 [PBS, Ut] or 6 [2G-H (CD28), *pCAR-H/T* or Trunc. CCR in pCAR] mice per group). Ut – untransduced. Day of CAR T-cell treatment is indicated by the arrow. Statistical analysis was performed using two-way ANOVA. ****$p<0.0001$; ***$p<0.001$; ** $p<0.01$; * $p<0.05$. (D) Bioluminescence images of mice at the specified time points are shown. *Related to Figure 5.*
